# Ligand-Directed Self-Assembly of Organic-Semiconductor/Quantum-Dot Blend Films Enables Efficient Triplet Exciton-Photon Conversion

**DOI:** 10.1021/jacs.4c00125

**Published:** 2024-03-08

**Authors:** Victor Gray, Daniel T. W. Toolan, Simon Dowland, Jesse R. Allardice, Michael P. Weir, Zhilong Zhang, James Xiao, Anastasia Klimash, Jurjen F. Winkel, Emma K. Holland, Garrett M. Fregoso, John E. Anthony, Hugo Bronstein, Richard Friend, Anthony J. Ryan, Richard A. L. Jones, Neil C. Greenham, Akshay Rao

**Affiliations:** † Cavendish Laboratory, University of Cambridge, J. J. Thomson Avenue, Cambridge CB3 0HE, U.K.; ‡ Department of Chemistry, Ångström Laboratory, Uppsala University, Box 532, SE-751 20 Uppsala, Sweden; § Department of Chemistry, The University of Sheffield, Sheffield S3 7HF, U.K.; ∥ Department of Materials, The University of Manchester, Engineering Building A, Booth Street East, Manchester M13 9PL, U.K.; ⊥ Cambridge Photon Technology, J. J. Thomson Avenue, Cambridge CB3 0HE, U.K.; # School of Physics and Astronomy, The University of Nottingham, University Park, Nottingham NG7 2RD, U.K.; ∇ John Owens Building, The University of Manchester, Oxford Road, Manchester M13 9PL, U.K.; ○ Yusuf Hamied Department of Chemistry, University of Cambridge, Lensfield Road, Cambridge CB2 1EW, U.K.; ◆ Center for Applied Energy Research, University of Kentucky, Research Park Drive, Lexington, Kentucky 40511, United States

## Abstract

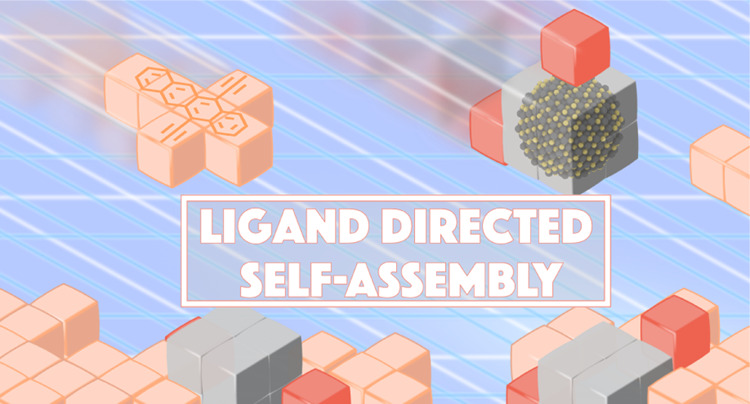

Blends comprising organic semiconductors and inorganic quantum dots (QDs) are relevant for many optoelectronic applications and devices. However, the individual components in organic-QD blends have a strong tendency to aggregate and phase-separate during film processing, compromising both their structural and electronic properties. Here, we demonstrate a QD surface engineering approach using electronically active, highly soluble semiconductor ligands that are matched to the organic semiconductor host material to achieve well-dispersed inorganic–organic blend films, as characterized by X-ray and neutron scattering, and electron microscopies. This approach preserves the electronic properties of the organic and QD phases and also creates an optimized interface between them. We exemplify this in two emerging applications, singlet-fission-based photon multiplication (SF-PM) and triplet–triplet annihilation-based photon upconversion (TTA-UC). Steady-state and time-resolved optical spectroscopy shows that triplet excitons can be transferred with near unity efficiently across the organic–inorganic interface, while the organic films maintain efficient SF (190% yield) in the organic phase. By changing the relative energy between organic and inorganic components, yellow upconverted emission is observed upon 790 nm NIR excitation. Overall, we provide a highly versatile approach to overcome longstanding challenges in the blending of organic semiconductors with QDs that have relevance for many optical and optoelectronic applications.

## Introduction

Organic semiconducting molecules and inorganic quantum dots (QDs) have found major applications in display technologies and are being widely studied for applications such as in photodetectors, photovoltaics, photocatalysis, sensors, and photon-frequency conversion (upconversion/downconversion). These material classes possess very different electronic properties, e.g., exciton binding energy, spin properties, mobilities, etc. There has been longstanding interest in combining them to form hybrid composites, to leverage these different properties, and to enable new device functionality.^1^ However, mixing two different types of materials comes with its own challenges, and to date, it has been difficult to prepare organic–inorganic QD blend films with high degrees of intermixing and efficient exciton or charge transfer across the organic–inorganic interface. This is because the components of organic semiconductor-quantum dot blends have a strong tendency to aggregate and phase-separate during processing due to a mismatch in their size, shape, and surface energies,^2−4^ with detrimental effects on device performance.^5−8^ Hence, deployment of these hybrid composites to applications such as photovoltaics (PV), photodetectors, and light-emitting diodes (LEDs) has not been particularly successful. In each of these areas, the underlying combination of organic and QD holds great promise, but blend morphology and poor interfaces have long compromised device performance.

**Figure 1:**
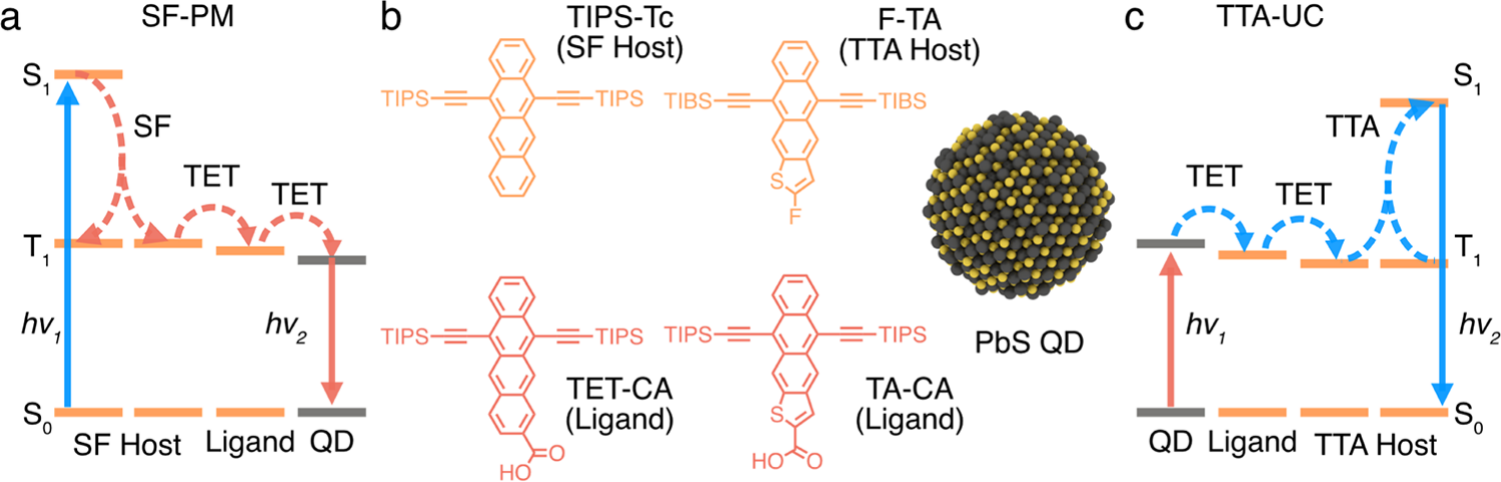
Schematic illustration of the singlet-fission photon multiplication (SF-PM) and triplet–triplet annihilation photon upconversion (TTA-UC) processes and used materials. (a) Jablonski diagram illustrating the flow of energy in the singlet-fission photon multiplication process: a high-energy photon (*h*ν_1_) is first absorbed by the SF host, followed by rapid singlet fission to generate two triplet excitons (T_1_). The triplet excitons are transferred (TET) in the host and later via a ligand to PbS quantum dots (QDs), which emit a photon (*h*ν_2_) when returning to the ground state. (b) Structures used in this study. TIPS-tetracene (TIPS-Tc) derivatives are used for SF photon multiplication, and thienoanthracenes (TAs) are used for TTA upconversion. Carboxylic acid anchoring groups are used to attach the ligands to the PbS QD surface. (c) TTA upconversion is the reverse process of SF-PM; absorption of a photon (*h*ν_1_) by two different PbS QDs is followed by triplet transfer via a ligand to the TTA host. When two triplet excitons in the host meet, they can fuse via TTA to generate a high-energy singlet state, leading to fluorescence of a high-energy photon (*h*ν_2_).

Here, we demonstrate a QD surface engineering approach using an electronically active, highly soluble semiconductor ligand that is matched to the organic host material, which allows us to direct the self-assembly of these blends, achieving optimal morphologies while preserving the electronic properties of both components as well as the interface between them. We demonstrate the effectiveness of this materials engineering approach to prepare organic–inorganic QD blends by applying it to two different photon-frequency conversion applications: singlet-fission-based photon multiplication (SF-PM) and triplet–triplet annihilation-based photon upconversion (TTA-UC), Figure 1.

Solid-state SF-PM and TTA-UC are both relevant to increasing the efficiency of solar cells and other solar harvesting devices,^9−13^ as well as applications in photocatalysis, imaging, etc. For example, TTA-UC, which fuses the energy of two low-energy photons to one photon of higher energy, can circumvent transmission losses in solar cells.^12,13^ SF-PM, on the other hand, has been suggested as an add-on technology for silicon photovoltaics (Si-PV) to overcome thermalization losses and increase device performance upward of 30%.^14^ However, to reach practical applications of SF-PM and TTA-UC materials, efficient solid-state materials have to be developed. In TTA-UC systems, a longstanding challenge is mixing the sensitizer, e.g., inorganic QDs, with an emissive annihilator molecule in the solid state.^15−21^ SF-PM being the reverse process of TTA-UC, splitting a high-energy photon into two low-energy photons faces similar challenges. We therefore find these two photon conversion processes suitable to demonstrate our materials engineering approach to achieve well-dispersed QDs in an organic film, minimizing aggregation-induced quenching of the QD and maintaining efficient triplet migration.

## Results and Discussion

### Dispersing QDs in Organic Hosts

**Figure 2:**
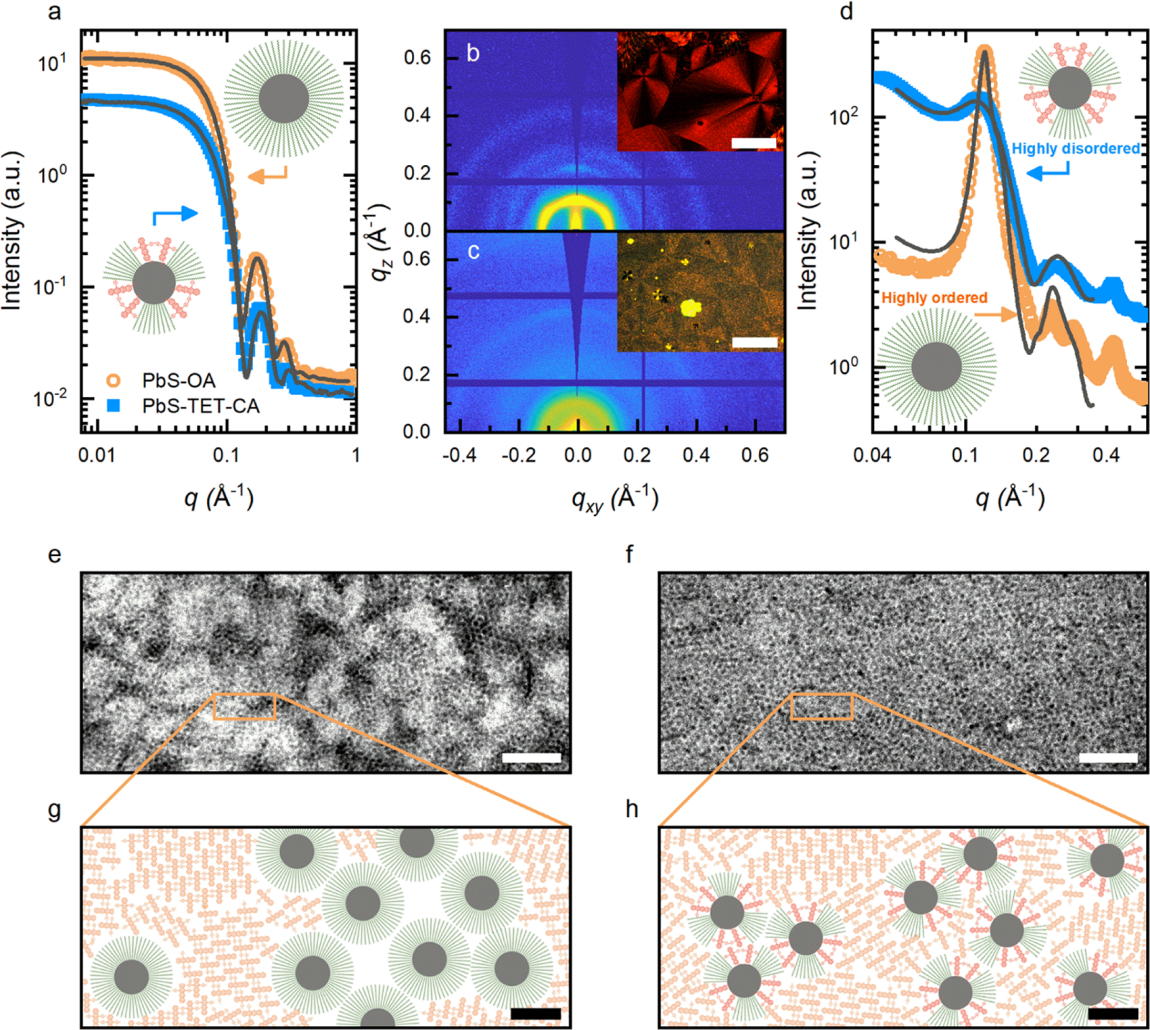
Ligand dependence of the PbS quantum dot dispersion within the singlet-fission host. (a) SANS data from before and after ligand exchange, i.e., PbS-OA (orange open circles) and PbS-TET-CA (blue closed squares), following subtraction of appropriate backgrounds, with associated fits to a core–shell sphere × hard sphere model (black curves). Insets: Schematic illustration of the population of ligands shifting from all OA (PbS-OA) to a mixture of OA and TET-CA (PbS-TET-CA). Two-dimensional grazing incidence X-ray scattering data for PbS-OA:TIPS-TC (b) and PbS-TET-CA:TIPS-Tc films (c). Insets showing polarized optical microscopy (POM) images (500 μm scale bar), with one-dimensional radially integrated data shown (d), with PbS-OA:TIPS-Tc (orange open circles), PbS-TET-CA:TIPS-Tc (blue closed squares), and associated fits to a face-centered cubic (FCC) colloidal crystal model (black curves). Transmission electron microscopy (TEM) (50 nm scale bar) for PbS-OA:TIPS-Tc (e) showing large aggregates (dark regions) within the SF host (lighter regions) and for PbS-TET-CA (f) showing a significantly more homogeneous quantum dot dispersion within the TIPS-Tc host. Illustration (5 nm scale bar) of the SF-PM structures for the highly ordered packing of the PbS-OA quantum dots (g) and the highly disordered dispersion of the PbS-TET-CA quantum dots (h) within the TIPS-Tc.

We start the discussion with the SF-PM system. As an SF host material, we chose 5,12-bis((triisopropylsilyl)ethynyl)tetracene (TIPS-Tc) (Figure 1b), a highly soluble, solution-processable molecular semiconductor shown to have an SF triplet yield of 130–180% in polycrystalline films.^22^ As TIPS-Tc has a T_1_ energy in the range of 1.1–1.2 eV, and triplet transfer to the quantum dot should be exothermic, PbS quantum dots with an exciton peak absorption at 1.08 eV were used as the inorganic acceptor (Supporting Figure 3). The photoluminescence quantum efficiency (PLQE) of the as-synthesized PbS quantum dots ligated with oleic acid (PbS-OA) was 31% in toluene. We recently showed that a tetracene-based ligand is necessary to achieve efficient triplet transfer in solution from TIPS-Tc to PbS quantum dots.^23^ The aliphatic organic ligands [e.g., oleic acid (OA)] that are bound to the surfaces of QDs following their synthesis inhibit the transfer of triplet excitons to the QDs in solution. As we develop below, these aliphatic organic ligands, which have been widely used in organic-QD blends to date, lead to phase segregation and QD aggregation upon film formation. Hence, PbS-OA quantum dots were ligand-exchanged with the “active-ligand”, 6,11-bis((triisopropylsilyl)ethynyl)tetracene-2-carboxylic acid (TET-CA), Figure 1b, to obtain TET-CA-ligated PbS quantum dots (PbS-TET-CA). In doing so, the quantum dot PLQE dropped slightly to 24% in toluene. The ligand exchange from PbS-OA to PbS-TET-CA was quantified by combining small-angle X-ray and neutron scattering measurements (SAXS and SANS, respectively). SAXS was employed to measure the PbS core radius and dispersity, with SANS providing insights into the changes in the quantum dot ligand envelope.^24^Figure 2a shows SANS data and associated fits, indicating that the ligand shell neutron scattering length density increases upon ligand exchange in a way that is consistent with a TET-CA ligand density of approximately 0.6 ± 0.1 ligands/nm^2^, which was confirmed via measurements of optical absorption. Further, analyzing the scattering length density and volume fractions of the ligand shell components, we conclude that significant TET-CA functionalization of the PbS quantum dot was achieved but that residual OA is also present. (For full details of the solution SAXS/SANS, see the Supporting Information Sections 2.1 and 2.2.)

SF-PM films were fabricated by blade coating solutions of TIPS-Tc (100 mg/mL) with either PbS-OA or PbS-TET-CA QDs (50 mg/mL) from toluene, allowing for film thicknesses ranging from 0.5 to 1 μm. Solution-casting TIPS-Tc by itself from low-volatility solvents generates highly crystalline, spherulitic-type morphologies. We find that blends of TIPS-Tc with the unmodified QDs (PbS-OA) form morphologies in which the QDs are highly aggregated, while QDs modified with the TET-CA ligand yield much more dispersed morphologies. Figure 2b,d shows grazing incidence small-angle X-ray scattering (GISAXS) from the QD:TIPS-Tc films, with clear structure factors between 0.05 and 0.35 Å^–1^, representing colloidal crystallization of aggregated quantum dots. Fits capturing the most significant features of the one-dimensional (1D) radially integrated scattering data were obtained for both PbS-OA:TIPS-Tc and PbS-TET-CA:TIPS-Tc films using a colloidal crystal model (see the Supporting Information for full fit parameters). The corresponding fit parameters for the PbS-OA:TIPS-Tc film, a lattice constant of 90.1 ± 3.9 Å and a lattice distortion factor of 0.08, indicate the formation of highly ordered QD aggregates. In contrast, the PbS-TET-CA blends show a much weaker colloidal ordering with fit parameters: a lattice constant of 76.8 ± 9.6 Å representing a decrease in the apparent thickness of TET-CA containing the ligand shell, and a greatly increased lattice distortion factor of 0.25 indicating a significantly enhanced contact between QDs and the singlet-fission host. The insets of Figure 2b,c show polarized optical microscopy (POM) images, indicating the presence of large radially orientated crystalline domains of the TIPS-Tc host, for both PbS-TET-CA:TIPS-Tc and PbS-OA:TIPS-Tc blend films. However, the nucleation density is much lower for the PbS-OA:TIPS-Tc films, suggesting that the PbS-TET-CA quantum dots are involved in the TIPS-Tc crystallization process.

The GISAXS data quantitatively show that PbS-OA QDs form highly ordered aggregate structures within TIPS-Tc, while PbS-TET-CA QDs are more randomly distributed within TIPS-Tc. This is further confirmed via TEM imaging shown in Figure 2e,f and Supporting Figures 4 and 5. Thus, the conventional aliphatic OA ligand, which has unfavorable interactions with the TIPS-Tc, leads to self-assembly processes during film formation, which gives rise to phase segregation and QD aggregation, as illustrated in Figure 2g. In contrast, the favorable interaction between the active TET-CA ligand and the bulk TIPS-Tc matrix allows for a directed self-assembly process, where phase segregation and QD aggregation are arrested, as illustrated in Figure 2h.

### Performance of Organic-QD Blends for SF-PM

**Figure 3:**
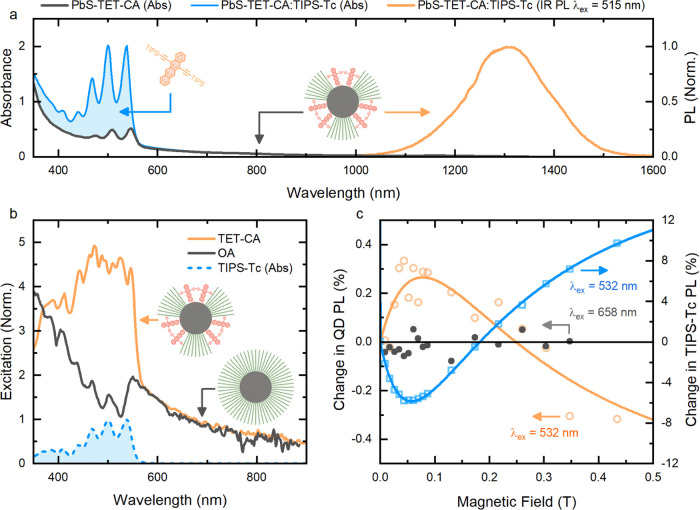
Absorbance and steady-state IR PL resulting from triplet harvesting in a film of TIPS-Tc and PbS-TET-CA quantum dots. (a) Absorbance (blue curve) and normalized IR PL (orange curve) of a PbS-TET-CA:TIPS-Tc thin film. For comparison, the absorbance of PbS-TET-CA quantum dots in toluene is represented by the black curve, with the difference highlighting the TIPS-Tc absorption (blue area). (b) QD IR PL excitation spectra of PbS-OA:TIPS-Tc (dark-gray curve) and PbS-TET-CA:TIPS-Tc films (orange curve), normalized to the average value between 650 and 700 nm. PbS QD emission was collected in the wavelength range 1300 ± 20 nm. Also plotted is the normalized absorbance spectrum of TIPS-Tc (dashed blue curve). (c) Percentage change in PL from the PbS-TET-CA QDs (orange and gray circles) and TIPS-Tc (blue squares) in a PbS-TET-CA:TIPS-Tc film as a function of an external magnetic field. The film was excited at either 532 nm (absorbed by SF and QD components) or 658 nm (selective excitation of the QD). Solid lines are guides to the eye for both the QD (orange curve) and TIPS-Tc (blue curve) PL change under 532 nm excitation.

We now turn to characterizing the optoelectronic properties of these films. The absorption and photoluminescence of the PbS-TET-CA:TIPS-Tc films are shown in Figure 3a. Figure 3b displays the IR PL excitation spectra of the QD:TIPS-Tc films. The reduction in IR PL for the PbS-OA:TIPS-Tc at excitation wavelengths where the TIPS-Tc is absorbing qualitatively shows that there is a relatively low triplet transfer from the TIPS-Tc to the PbS-OA QDs. In contrast, excitation spectra of PbS-TET-CA:TIPS-Tc films show high levels of energy transfer from TIPS-Tc to the PbS QDs, which we show below arise from triplet excitons.

To quantitatively evaluate the photon multiplication performance in the films, we measure the PLQE when exciting the SF host material TIPS-Tc (at 515 nm) and compare it to direct excitation of the PbS-TET-CA QDs (at 658 nm). The PLQE increases from (15.4 ± 1.0)% (658 nm, exciting only QDs in the blend) to (24.5 ± 1.0)% (515 nm, exciting both components of the blend). This enhancement of (59 ± 12)% suggests efficient SF followed by triplet energy transfer (TET) to the emissive QDs. Using the relative absorption in the PbS-TET-CA quantum dots and the SF host, an exciton multiplication factor (EMF) of η_EMF_ = (186 ± 18)% can be estimated from eq S9; η_EMF_ is also given by the product of the singlet-fission yield η_SF_ and the triplet transfer efficiency η_TET_. η_EMF_ serves as a metric of blend performance as it captures the retention of SF properties of the SF host material, the quality of morphology via the efficient diffusion of triplets to the QDs, and the quality of the interface via the transfer of the triplets into the QD. In contrast to the PbS-TET-CA:TIPS-Tc films, films of PbS-OA:TIPS-Tc show a drop in PLQE when the SF host is excited, from (17.2 ± 1)% (658 nm, exciting only QDs in the blend) to (3.8 ± 1)% (515 nm, exciting both components of the blend), as shown in Table 1.

**Table 1:** Photoluminescence Performance for Films of PbS Quantum Dots in a TIPS-Tc SF Host^a^

quantum dot	PLQE (λ_ex_ = 515 nm)	PLQE (λ_ex_ = 658 nm)	η_EMF_
PbS-OA	(3.8 ± 1)%	(17.2 ± 1)%	(−4 ± 8)%
PbS-TET-CA	(24.5 ± 1.0)%	(15.4 ± 1.0)%	(186 ± 18)%

a The PLQE of the PbS QD emission in films of PbS-OA:TIPS-Tc and PbS-TET-CA:TIPS-Tc under excitation at either 515 nm (absorbed by both SF and QD components) or 658 nm (absorbed by QD only). Based on these PLQE values and the relative absorption of the film components, the exciton multiplication factor, η_EMF_, is calculated.

To verify that the PLQE enhancement originates from SF and triplet transfer, we perform magnetic-field-dependent PL measurements (Figure 3c). We observe an initial decrease in TIPS-Tc PL at low magnetic fields (<150 mT) followed by an increase at higher magnetic fields. This behavior is typical for SF materials.^10,23^ The quantum dot PL shows the opposite trend, demonstrating that the QD PL arises from triplet energy transfer to the QDs. Transient PL and transient absorption measurements further support the conclusion that the PL enhancement in the PbS-TET-CA:TIPS-Tc blends arises due to efficient SF in TIPS-Tc (η_SF_ = (192 ± 28)%), quantitative triplet transfer to the QD (η_TET_ = (97 ± 11)%), followed by QD emission (see the Supporting Information for details). Transient absorption on time scales from picoseconds to microseconds also supports the conclusion of SF-enhanced QD PL and suggests a two-step transfer mechanism via the organic ligand (see Section 8 in the Supporting Information), as previously reported in solution.^25^

### Organic-QD Blends for TTA-UC

With the successful demonstration of SF-PM films, we expand our approach to TTA-UC films. As seen in Figure 1a, the difference in the SF-PM and TTA-UC systems is the direction of the flow of energy. We therefore prepared 1.6 eV PbS QDs with 5,10-bis((triisopropylsilyl)ethynyl)anthra[2,3-*b*]thiophene-2-carboxylic acid ligands (PbS-TA-CA). The TA-CA ligand could not be prepared in the same manner as the TET-CA ligand as we found that previous reports on the synthesis of the brominated quinone yielded the wrong product. Instead, we prepared the thienoacene ligand via simple deprotonation and carboxylation using LDA (see the Supporting Information for details). For the annihilator and organic host, we chose the structurally similar ((2-fluoroanthra[2,3-*b*]thiophene-5,10-diyl)bis(ethyne-2,1-diyl))bis(triisobutyl-silane) (F-TA) as the annihilator (*S*_1_ ∼ 2.48 eV and *T*_1_ ∼ 1.35 eV).

Using PbS-TA-CA as triplet sensitizers in a toluene solution with F-TA as annihilators resulted in little TET and inefficient TTA-UC, likely due to a mismatch in *T*_1_ energies (see the Supporting Information Section 9 for details). With rubrene (*T*_1_ = 1.14 eV^26^) TET in solution was quantitative and resulted in a measurable upconversion quantum yield (UCQY) of 0.7% for PbS-TA-CA. However, for solid-state films, rubrene is not ideal for a number of reasons: (i) emission quenching is common in the solid state due to SF,^27^ and (ii) the molecular structure is significantly different from the TA-CA ligand, which limits the favorable interactions driving the desired self-assembly of QDs in the host. This is corroborated by findings from studies of rubrene:PbS-TET-CA films (as shown in Supporting Figure 1), which show that a rubrene:QD film forms a morphology where the QDs form a highly ordered close-packed monolayer and are not distributed within the bulk of the rubrene. Rubrene has a further disadvantage of being sparingly soluble in common organic solvents, providing further processing challenges. On the other hand, F-TA was shown to have a relatively high PLQY in the solid state (50 ± 5% in the neat film), has a better-matched molecular structure to TA-CA, and is highly soluble in common organic solvents. We therefore chose to study the self-assembly of PbS-TA-CA in F-TA films (PbS-TA-CA:F-TA).

Upconversion films were prepared by blade coating solutions containing 100 mg/mL F-TA hosts and 10 mg/mL PbS-TA-CA. The PbS-TA-CA:F-TA films formed morphologies where QDs are not as dispersed in a single arrangement throughout the film, as observed for the PbS-TET-CA:TIPS-Tc films, but are instead arranged in two distinct populations: (i) as well-dispersed QDs distributed throughout the film and (ii) as aggregated QDs. Thus, the colloidal crystal model alone could not adequately fit the experimental scattering data. Instead, a model taking into account the two QD populations in the film was employed. This sphere × hard sphere + colloidal crystal model is separated into the component parts in Supporting Figure 2, showing the contributions to the model from both the dispersed and aggregated QD fractions and the full fit parameters. From the model fits, an estimate of the volume fraction of the scattering material in either structure was obtained, and the fraction of dispersed QDs within the film was estimated to 0.61 [full fit parameters and further scattering details are available in the Supporting Information].

The differences in the QD dispersibility between the PbS-TET-CA:TIPS-Tc and PbS-TA-CA:F-TA blend films most likely arise from slight changes in the crystallization behavior of the TIPS-Tc and F-TA films, with the crystallization of F-TA likely enhanced due to F–F and F–S interactions, as well as the known interactions between fluorinated and nonfluorinated aromatic surfaces.^31^ A similar behavior was previously observed when changing the organic matrix; however, such studies were limited to a single “matched” QD ligand. The approach demonstrated here shows that effective matching between the organic host matrix and the QD ligand is critical for effectively dispersing QDs within an organic host.

**Figure 4:**
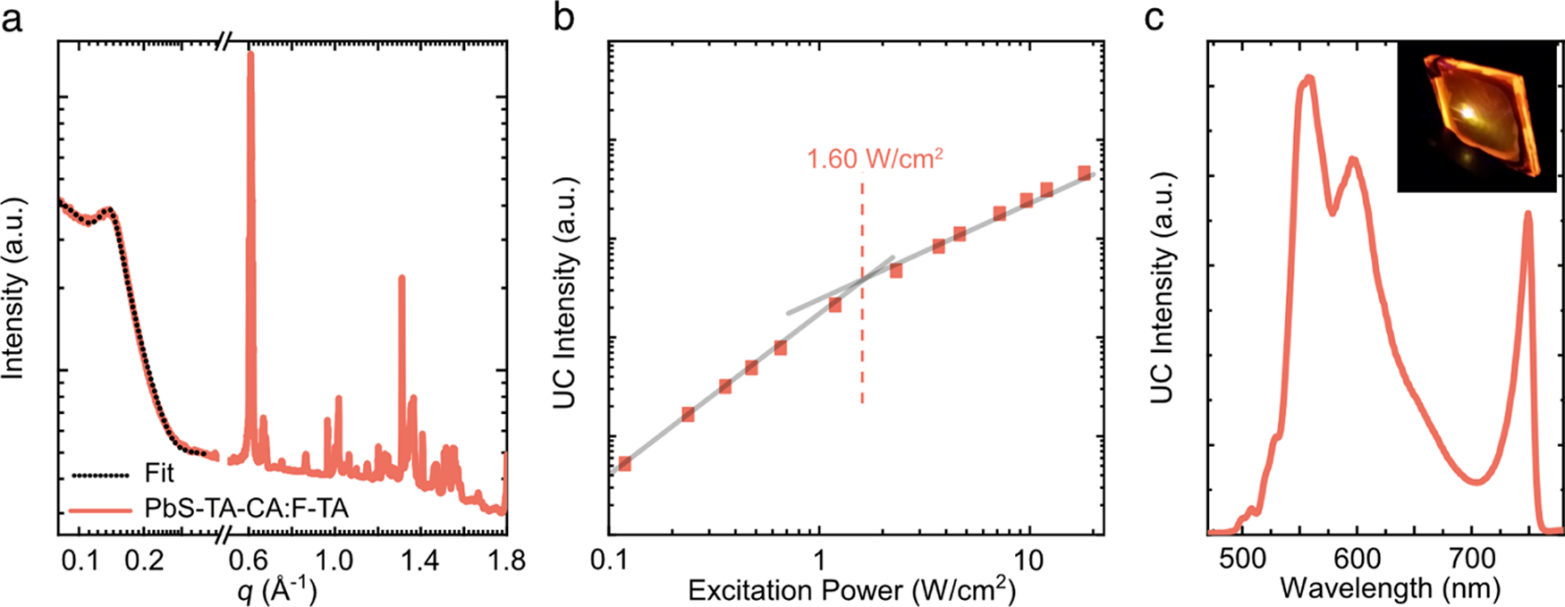
(a) One-dimensional grazing incidence X-ray scattering data for PbS-TA-CA:F-TA films cast at 10 mg/mL with associated fit to the QD scattering region (0.08–0.4 Å^–1^) using a sphere × hard sphere + FCC colloidal crystal model (dotted black curve). (b) Excitation intensity dependence of the upconversion film comprising the F-TA host and PbS-TA-CA sensitizer QDs. Also illustrated is the determined threshold intensity and the linear fit for the low- and high-intensity regions. (c) Recorded upconverted emission of the 10 mg/mL QD-loaded PbS-TA-CA:F-TA films upon excitation with 790 nm photons. The inset shows a photograph of the yellow emission upon 790 nm excitation.

The optical properties of the PbS-TA-CA:F-TA films were investigated. The PLQY of the F-TA host upon direct excitation was 17.8 ± 1.1%, indicating about 65% quenching compared to the neat film. Such quenching can be due to singlet energy transfer and reabsorption from the F-TA host to QD, as observed in other upconversion films.^28,29^ These issues could be overcome by adding a singlet collector.^28,29^ Upon excitation of the QDs (790 nm), bright-yellow upconverted emission from F-TA is observed by the naked eye (Figure 4c). The upconversion quantum yield (UCQY) was determined to be 0.1 ± 0.06%, which is in line with many other solid-state UCQYs.^15,17,18,20,30^ Interestingly, compared to F-TA in solution, the UCQY is significantly enhanced in the solid state. An increase in UCQY in the solid state is an unusual behavior as quenching when going from solution to the solid state is a significant challenge for solid-state systems.^15,17,18,20^ We consider that the main reason for the low yield in the solid (and solution) state stems from the fact that the triplet energy of F-TA likely lies slightly above the triple energy of TA-CA, as discussed in detail in the Supporting Information and supported by the difference in singlet energies seen in the films (Supporting Figure S23.). This misalignment thus reduces the efficiency of triplet migration from PbS to the F-TA host.

## Conclusions

In summary, we have demonstrated a route to overcoming the longstanding problems of phase segregation and aggregation in solution-processed organic-QD blends. From our spectroscopic and morphological characterization, we can conclude that the role of the QD ligand is 2-fold; it enables efficient triplet transfer between the host and the QDs and plays a key role in altering the surface chemistry of the QDs, thereby achieving a directed self-assembly process, which allows both optimal morphology while preserving electronic properties of both components as well as the interface between them. In the SF-PM films fabricated with PbS-TET-CA:TIPS-Tc, efficient SF (η_SF_ = 192 ± 28%) in the TIPS-Tc is followed by efficient triplet energy transfer to the QDs (η_TET_ = 97 ± 11%), giving rise to a ∼190% exciton multiplication factor (out of a possible 200%). In comparison, films fabricated with conventional aliphatic ligands show strong QD aggregate formation and very poor exciton harvesting. The same approach was also applied to solid-state photon upconversion; despite a mismatch in energy alignment between the TA-CA ligand and the annihilator host material F-TA, a measurable solid-state UCQY was observed (0.1 ± 0.06%). In contrast, the same system in the solution phase showed very inefficient UC.

Our results provide a clear route for future work, where optimizing the relative energy of the organic and inorganic materials will allow development of solid-state SF-PM composites relevant for Si-PV enhancement and TTA-UC composites with higher UCQYs. Specifically, QDs with higher PLQYs must be developed for SF-PM to be practical. However, PbS QDs with larger band gaps (PL around 1000 nm) can be synthesized, with PLQYs approaching 80%. Thus, a more pressing issue is to develop efficient SF materials with triplet energies that can be combined with such QDs.^25^ For UC films, organic materials with higher PLQYs in the solid state are also required to increase the overall UCQY.

More broadly, our results offer a general strategy to control the morphology for a range of applications where organic-QD blends are desired. For instance, as described herein, photon management devices based on organic-QD composites, which have so far been limited to solution phase or bilayers; or LEDs and PV based on QDs within an organic host; as well as new types of devices relying on organic-QD composites, such as spin memories, would all benefit from our strategy to avoid aggregation-induced quenching effects.

## Data Availability

The data that support the findings of this study are openly available in Apollo—University of Cambridge Repository at https://doi.org/10.17863/CAM.106622.

## Supporting Information Available

Experimental details, materials, and methods, and additional supporting experimental data including grazing incidence X-ray scattering, TEM images, picosecond transient absorption, IR transient photoluminescence, and description of the modeling of kinetic and scattering data (PDF)
