# Supplementary material for: Ligand-Directed Self-Assembly of Organic-Semiconductor/Quantum-Dot Blend Films Enables Efficient Triplet Exciton-Photon Conversion
Source: J Am Chem Soc. 2024 Mar 8;146(11):7763–70. doi: 10.1021/jacs.4c00125 (PMC10958494; doi:10.1021/jacs.4c00125)
Supplement: Supplementary file 1 — ja4c00125_si_001.pdf [file ja4c00125_si_001.pdf]

Supplementary Information: Ligand Directed Self-Assembly of Organic-Semiconductor/Quantum-Dot Blend Films Enables Efficient Triplet Exciton-Photon Conversion

Victor Gray[a,b]†, Daniel T. W. Toolan[c,d]†, Simon Dowland[e]†, Jesse Allardice[a]†, Michael P. Weir[f]†, Zhilong Zhang[a], James Xiao[a], Anastasia Klimash[h], Jurjen F. Winkel[e], Emma K. Holland[i], Garrett M. Fregoso[i], John Anthony[i], Hugo Bronstein[h], Richard Friend[a], Anthony J. Ryan[c], Richard A. L. Jones[g], Neil C. Greenham[a] and Akshay Rao[a]*

Contacts

[a]Cavendish Laboratory, University of Cambridge, J. J. Thomson Avenue, Cambridge, CB3 0HE, UK

[b]Department of Chemistry, Ångström Laboratory, Uppsala University, Box 532, SE-751 20 Uppsala, Sweden

[c]Department of Chemistry, The University of Sheffield, Sheffield S3 7HF, United Kingdom

[d]Department of Materials, The University of Manchester, Engineering Building A, Booth Street East, Manchester, M13 9PL, UK

[e]Cambridge Photon Technology, J. J. Thomson Avenue, Cambridge, CB3 0HE, UK

[f]School of Physics and Astronomy, The University of Nottingham, University Park, Nottingham, NG7 2RD, UK.

[g]John Owens Building, The University of Manchester, Oxford Road, Manchester M13 9PL, UK.

[h]Yusuf Hamied Department of Chemistry, Lensfield Road, Cambridge, CB2 1EW, UK

[i]Center for Applied Energy Research, University of Kentucky, Research Park Dr., Lexington KY 40511, USA

†: Authors contributing equally to this work.

*: Corresponding Author, Akshay Rao, e-mail: ar525@cam.ac.uk

1 Contents

1 Contents 2
2 Methods 4
2.1 Small-Angle Neutron Scattering 4
2.2 Grazing Incidence X-ray Scattering 5
2.3 TEM 9
2.4 Ligand Exchange 9
2.5 Steady-State Absorption 10
2.6 PLQE and Excitation Scan of SF-PM films 10
2.7 Transient Absorption 10
2.8 IR TCSPC 11
2.9 Steady-State and Magnetic Dependent PL 11
2.10 Synthesis of TA-CA Ligand 11
2.11 Photon Upconversion Measurements 14
2.11.1 Upconversion in Solution 15
2.11.2 Upconversion in Films 15
3 TEM of SF-PM films 15
4 Extracting an Exciton Multiplication Factor for SF-PM films 17
5 Absorbance Spectra of SF-PM film 18
6 Magnetic Field Dependent PL of SF-PM films 18
7 Picosecond Transient Absorption of SF-PM films 18
8 Nanosecond Transient Absorption and IR Transient PL of SF-PM films 19
8.1 Nanosecond Transient Absorption (ns-TA) 19
8.2 Analysis of the QD GSB Shifting 23
8.3 Kinetic Parameters Extracted from nsTA 24
8.4 IR Transient PL (IR TCSPC) 24
8.5 Triplet Flux Deconvolution 27
8.6 Triplet Transfer Models 28
8.6.1 Two-Species Model 28
8.6.2 Three-Species Model 31

9 Upconversion 33
9.1 Energy Levels of Anthrathiophenes 33
9.2 Triplet Energy transfer in solution 34
9.3 Upconversion in solution 36
9.4 Upconversion in Film 37
10 References 37

2 Methods

2.1 Small-Angle Neutron Scattering

SANS was carried out on the SANS2D[1] small-angle diffractometer at the ISIS Pulsed Neutron Source (STFC Rutherford Appleton Laboratory, Didcot, U.K.).[2] Samples before (PbS-OA) and after ligand exchange (PbS-TET-CA) were prepared in deuterated toluene, providing the necessary contrast, and were contained in 2 mm path length quartz cells (Hellma GmbH). In the following, the magnitude of the scattering vector is defined as $q = \frac{4\pi sin\theta}{\lambda}$ where 2θ is the angle between the incident and scattered X-ray or neutron of wavelength. A simultaneous *q*-range of 0.006 – 1.2 Å^{-1} was achieved utilizing an incident wavelength range of 1.65 – 16.5 Å and employing an instrument set up of L1 = L2 = 4 m, where L1 and L2 are the pre and post sample flightpaths respectively, with the rear detector offset vertically 75 mm and horizontally 100 mm. The beam diameter was collimated to 12 mm at the sample. For all collected data, each raw scattering data set was corrected for the detector efficiencies, sample transmission and background scattering and converted to scattering cross-section data (∂Σ/∂Ω vs. *q*) using the instrument-specific software.[3] These data were placed on an absolute scale (cm^{-1}) using the scattering from a standard sample (a solid blend of hydrogenous and perdeuterated polystyrene) in accordance with established procedures.[4]

Appropriate backgrounds were subtracted from each sample, namely oleic acid in d-toluene for PbS-OA[5] and TIPS-tetracene in d-toluene for PbS-TET-CA, to approximate the scattering from residual TET-CA in solution following ligand exchange. Fitting was performed using the *SasView* software package.[6] The data were fitted to a core-shell sphere model with a hard-sphere structure factor. The core-shell sphere model takes into account the scattering from the spherical core and a single spherical shell, so that the scattering intensity (when multiplying by the appropriate structure factor) is given by

$$I(q) = \frac{scale}{V} F^2(q) S(q) \tag{S1}$$

where

$$F(q) = 3\left[V_c(\rho_c - \rho_s)\frac{\sin(qr_c) - qr_c\cos(qr_c)}{(qr_c)^3} + V_s(\rho_s - \rho_{solv})\frac{\sin(qr_s) - qr_s\cos(qr_s)}{(qr_s)^3}\right]^2 \tag{S2}$$

and V_c and V_s are the volumes, r_c and r_s are the radii, and ρ_c and ρ_s are the scattering length densities of the core and shell respectively, and ρ_{solv} is the scattering length density of the solvent. $S(q)$ is, as stated, the hard sphere structure factor (as implemented in *SasView* version 4.2.2) which is a calculation of the interparticle structure factor for monodisperse spherical particles interacting through excluded volume interactions. This is calculated using the Percus-Yevick closure[7] where the inter-particle potential is given as:

$$U(r) = \begin{cases} \infty & r < 2R \\ 0 & r \geq 2R \end{cases} \tag{S3}$$

The core radius of 16.3 Å and lognormal polydispersity of 0.1 were used as constraints to the SANS fitting, detailed procedures for the characterisation of this PbS-OA sample set is described elsewhere.[5] The main

output of the model was therefore a scattering length density, thickness, and polydispersity of the ligand shell. In all cases, the component scattering length densities are taken as ρ_{OA} = -0.24 (packed OA tails), ρ_{TET-CA} = 0.9 and $\rho_{d-toluene}$ = 5.68 × 10^{-6} Å^{-2}. In the case of PbS-OA, the shell SLD is simply given by $\rho_{shell} = \phi_{OA}\rho_{OA} + \phi_{solvent}\rho_{solvent}$ allowing simple calculation of the respective volume fractions within the shell of oleic acid and solvent as ϕ_{OA} = 0.83 and $\phi_{solvent}$ = 0.17. In the case of PbS-TET-CA, a ligand density on the order of 0.6 ± 0.1 ligands/nm^2 is typically measured by absorption. The exchanged ligand shell is therefore described by $\rho_{shell} = \phi_{TET-CA}\rho_{TET-CA} + \phi_{OA}\rho_{OA} + \phi_{solvent}\rho_{solvent}$. For a TET-CA ligand density of 0.6 ± 0.1 ligands/nm^2, this is consistent with values of ϕ_{TET-CA} = 0.35, ϕ_{OA} = 0.42 and $\phi_{solvent}$ = 0.23, leading to the conclusion that significant TET-CA functionalisation of the PbS has been achieved, but that residual OA is also present.

2.2 Grazing Incidence X-ray Scattering

Grazing incidence X-ray scattering measurements were carried out on two instruments Xeuss 2.0 instrument equipped with an Excillum MetalJet liquid gallium X-ray source for TIPS-Tc:PbS-TET-Ca and Rubrene:PbS-TET-CA films and at I07 (Diamond Light Source, Rutherford, U.K.) for F-TA-PbS-TACA films. The Xeuss 2.0 instrument equipped with an Excillum MetalJet liquid gallium X-ray source. Alignment was performed on silicon substrates via three iterative height (*z*) and rocking curve (Ω) scans, with the final grazing incidence angle set to Ω = 0.2° and collimating slits of 0.25 × 0.3 mm ("high resolution" mode) were employed. Scattering patterns for TIPS-Tc:PbS-TET-CA films were recorded on a vertically-offset Pilatus 1M detector with a sample to detector distance of 559 mm, calibrated using a silver behenate standard to achieve a *q*-range of 0.045 – 1.2 Å^{-1}. Scattering patterns for rubrene:PbS-TET-CA films were recorded on a vertically offset Pilatus 1 M detector with a sample-to-detector distance of 332 mm, calibrated using a silver behenate standard to achieve a *q*-range of 0.045–1.5 Å^{-1}. Two-dimensional images were recorded with exposure times of 900 and 600 s for the reseective TIPS-Tc:PbS-TET-CA and rubrene:PbS-TET-CA films. Data reduction was performed using GISAXS-GUI MATLAB toolbox.[8] Two-dimensional scattering data was reduced to one-dimensional via radial integration, which was performed with a mask to remove contributions from "hot pixels", substrate horizon and the reflected beam. Fitting was performed using the *SasView* software package.[6]

F-TA-PbS-TA-CA films were prepared on silicon and GIWAXS was performed at I07 (Diamond Light Source, Rutherford, U.K.) at an X-ray beam energy of 12.4 keV. Scattering patterns were recorded on a vertically-offset Pilatus 2M detector with a sample to detector distance of 635.62 mm, calibrated using a silver behenate standard to achieve a Q range of 0.045 – 1.8 Å^{-1}. For scattering data collected using either instrument, alignment was performed via three iterative height (z) and rocking curve (Ω) scans, with the final grazing incidence angle set to Ω = 0.3°. The two-dimensional scattering patterns were masked to remove the sample horizon, detector module gaps and beam-stop and radially integrated from the apparent beam centre. Data correction and reduction was performed using the GIXSGUI MATLAB toolbox.[1] Two-dimensional scattering data was reduced to one-dimensional via radial integration, which was performed with a mask to remove contributions from "hot pixels", the substrate horizon and reflected beam.

TIPS-Tc:PbS-OA and TIPS-Tc:TET-CA data were fitted to *Face-centred cubic (FCC) lattice with paracrystalline distortion.* The FCC paracrystal model (as implemented in *SasView* version 4.2.2) calculates the scattering from a face-centred cubic lattice with paracrystalline distortion. With the scattering intensity $I(q)$ calculated as:

$$I(q) = \frac{scale}{V_p} V_{lattice} F(q) Z(q) + background \quad \text{(S4)}$$

where scale is the volume fraction of spheres, V_p is the volume of the primary particle, $V_{lattice}$ is a volume correction for the crystal structure, $F(q)$ is the form factor of the sphere (normalized), and $Z(q)$ is the paracrystalline structure factor for a face-centered cubic structure.

The lattice correction (the occupied volume of the lattice) for a face-centred cubic structure of particles of radius R and the nearest neighbour separation D is

$$V_{lattice} = \frac{16\pi}{3} \frac{R^3}{\left(D\sqrt{2}\right)^3} \quad \text{(S5)}$$

The distortion factor (one standard deviation) of the paracrystal is included in the calculation of $Z(q)$

$$\Delta a = \mathrm{gD} \quad \text{(S6)}$$

where g is a fractional distortion based on the nearest neighbour distance.

Table S1: *Fit parameters for TIPS-Tc:PbS-OA and TIPS-Tc:PbS-TET-CA scattering data.*

	TIPS-Tc:PbS-OA	TIPS-Tc:PbS-TET-CA
scale	0.609	0.544
background	0.151	3.034
Lattice constant	89.7	76.8
Lattice order parameter	0.086	0.250
PbS core radius (Å)	22.34	22.34
PbS core log normal polydispersity (Å)	0.080	0.075
PbS X-ray sld(Ga) (10^{-6} Å^{-2})	50.7	50.7
TIPS-Tc X-ray sld (10^{-6} Å^{-2})	10.2	10.2

Rubrene:PbS-TET-CA solutions were prepared at 10:2.5 mg mL^{-1}, respectively, in toluene were heated to 50 °C for 1 h and vortex-mixed prior to use. Silicon substrates were cleaned with Decon and ethanol followed by three deionized water rinses. Fifty microliters of casting solutions was deposited on silicon substrates and spun-cast at 1500 rpm for 2 min. The two dimensional grazing incidence X-ray scattering data for PbS-TET-CA:Rubrene films is shown in Supplementary Figure 1.

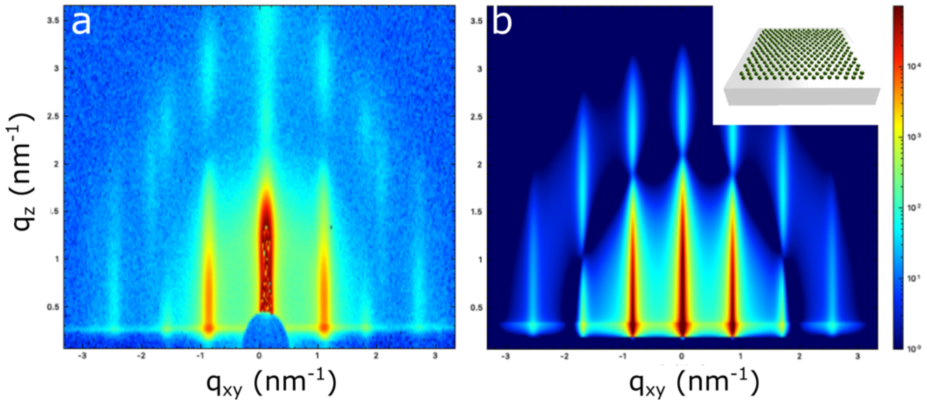

Supplementary Figure 1*.* ***(a)*** *Two dimensional grazing incidence X-ray scattering data for PbS-TET-CA:Rubrene film blade coated from 2.5:10 mg/ml toluene.* ***(b)*** *Simulated grazing incidence scattering data for a ordered monolayer of QDs on the substrate surface, with corresponding real-space representation of this structure shown as an insert [generated using BornAgain software using a 2D interference lattice.[9]]*

Scattering data for F-TA:PbS-TA-CA blend is shown in Supplementary Figure 2. This data could not be fitted to a single FCC paracrystal model as for the TIPS-Tc:QD blends. A model was fitted to the data comprising a spherical form factor with a hard-sphere structure factor that describes scattering from QDs still distributed with liquid-like order within the film, in addition to a FCC paracrystal contribution to take into account regions of the film where the QDs are close-packed into a colloidal crystal.

The expression of $I(q)$ for this sphere×hard-sphere+FCC model is given as:

$$I(q) = F(q)S(q) + \frac{scale}{V_p} V_{lattice} F(q) Z(q) + background \quad \text{(S7)}$$

where $F(q)$ is the form factor of the sphere (normalized) and $S(q)$ is the hard sphere structure factor that multiplies the form factor for dispersed scatterers at each point (and is equivalent in implementation in SAXS as it is in SANS; see Section 2.1 above for additional detail).

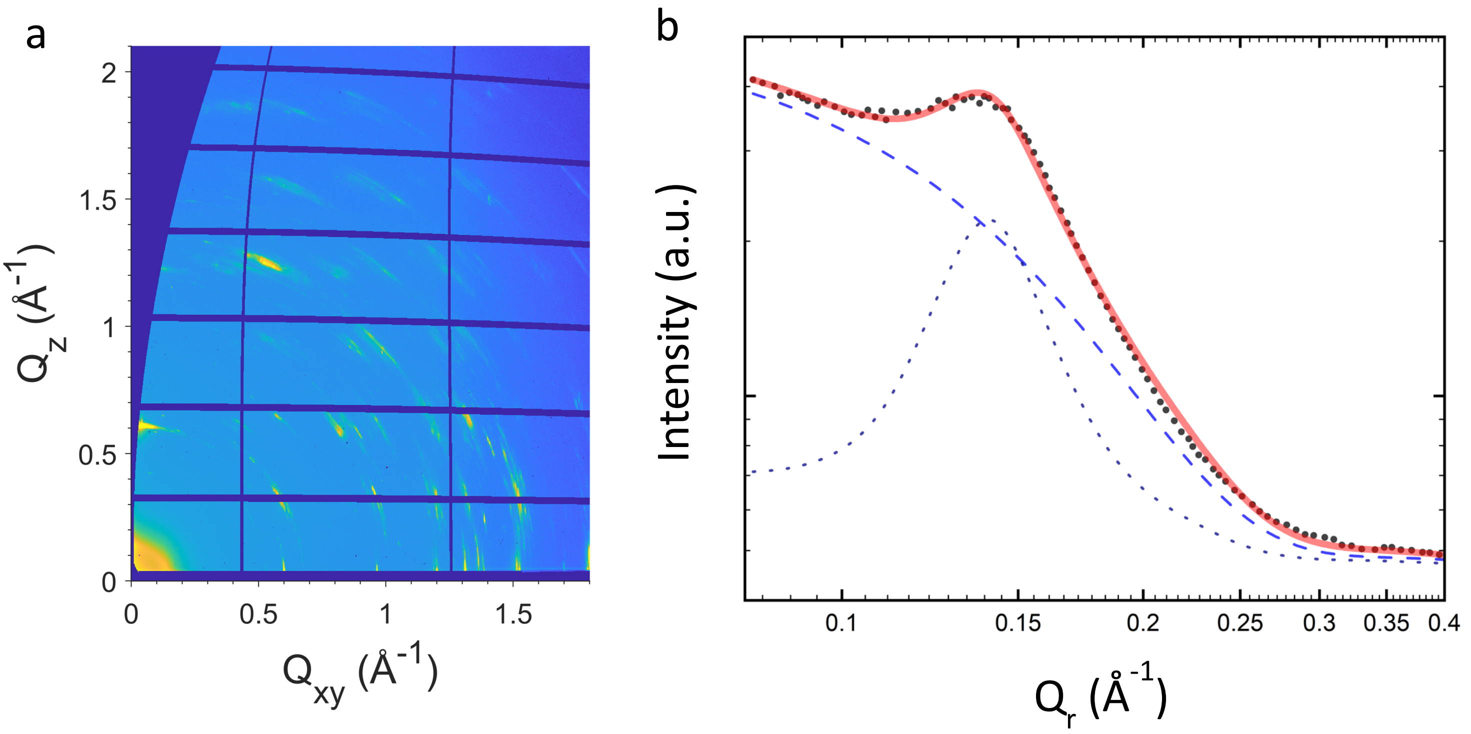

Supplementary Figure 2*. Two dimensional grazing incidence X-ray scattering data for PbS-TA-CA:F-TA* ***(b)****, (black circles) associated fits to an sphere*hard-sphere + FCC colloidal crystal model (red curves, with the separate sphere*hard-sphere (blue dashed line) and FCC colloidal crystal (small navy dots) components of the model fit.*

Table S2*: Fit parameters for PbS-TACA:F-TA films scattering data.*

	F-TA:PbS-TA-CA
	PbS-TACA (10 mg/ml)
scale	67.46
background	461.80
PbS X-ray sld(Ga) (10^{-6} $Å^{-2}$)	50.70
TIPS-Tc X-ray sld (10^{-6} $Å^{-2}$)	10.20
PbS core radius (Å)	13.50

PbS core log normal polydispersity (Å)	0.17
Sphere volume fraction	0.00
FCC scale factor	0.32
Lattice constant	74.30
Lattice order parameter	0.16

The volume fraction of scattering material in either sphere*hard sphere or FCC colloidal crystal was obtained from a limited-range calculation (i.e. performed on only the experimental *q*-range without extrapolation) of the scattering invariant $Q^*_{limited} = \int_{q_{min}}^{q_{max}} q^2 I(q)\, dq$ for each of the respective model components.

2.3 TEM

Transmission electron microscopy to investigate film morphology was performed using an FEI Tecnai F20 at 200 kV accelerating voltage. Film samples at 50 mg mL^{-1} QDs and 100 mg mL^{-1} TIPS-Tc were transferred onto 200-mesh Cu grids (Agar AGS160).

2.4 Ligand Exchange

Synthesis of PbS QDs was carried out following the procedure by Hines and Scholes with modifications[10,11] In summary, PbO (0.45 g), oleic acid (8 g) and 1-octadecene (10 g) were degassed in a three-necked flask at 110 °C for 2 h. The temperature was then reduced to 95 °C. Under nitrogen, a solution of bis(trimethylsilyl)sulphide (210 µL) in 1-octadecene (5 mL) was rapidly injected into the lead precursor solution. After cooling naturally to room temperature, the PbS QDs were washed 4 times by precipitation/re-dispersion with acetone and hexane. The purified QDs were stored in a nitrogen filled glovebox at high concentration (>40 mg mL^{-1} / >100 µM) until use. Ligand exchange was carried out under nitrogen. The QDs in toluene were diluted to 8 mg mL^{-1} in a Toluene/THF mixture of 4:1. The ligand in 100 mg mL^{-1} THF solution was added to the QD solution, keeping a ligand to QD mass ratio of 1:1 for TET-CA and 1:2 for TA-CA (for medium and high coverage 1:1 and 2:1ratios were used, respectively).

Ligand coverage was estimated from UV/Vis absorption using the molar absorption coefficient of the TET-CA ligand as 25500 M^{-1}cm^{-1} at the peak absorption in toluene. The ligand area was used assuming the QD as a sphere with a diameter estimated from the empiric formula in Moreels *et al.*[12]

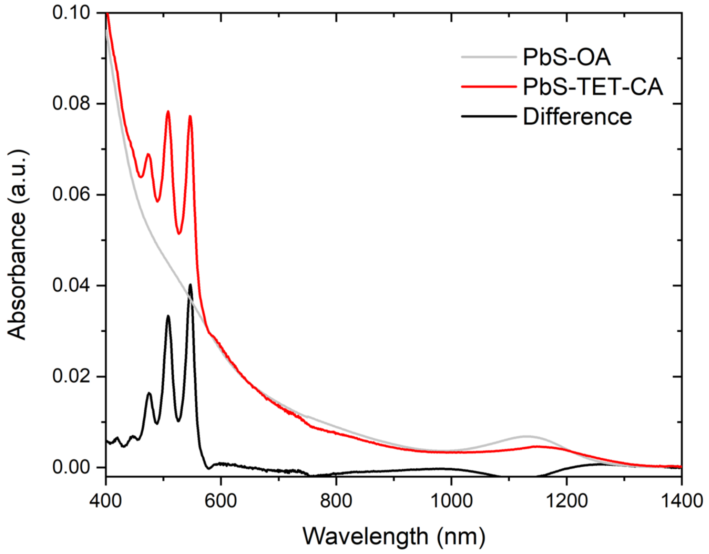

Supplementary Figure 3*: Absorption of PbS-OA, PbS-TET-CA after ligand exchange and difference spectra used to calculate the ligand coverage.*

2.5 Steady-State Absorption

A Shimadzu UV-3600Plus spectrometer was used to measure the absorbance spectra of the solutions and films.

2.6 PLQE and Excitation Scan of SF-PM films

The integrating sphere and PLQE measurement procedure has been described previously.[13,14] In summary, an integrating sphere with a Spectralon-coated interior (Newport 819C-SL-5.3) was used. 515 nm (2.9 x 10^{15} photons $s^{-1}cm^{-2}$ at the sample) and 658 nm (1.8 x 10^{16} photons $s^{-1}cm^{-2}$ at the sample) laser diodes (Thorlabs) with a beam diameter at the sample of 3 mm was used as the excitation source. Light from the sphere was coupled into an Andor Kymera 328i Spectrograph equipped with an InGaAs detector (Andor, iDus InGaAs 490). From the reproducibility of consecutive measurements, we estimate the absolute uncertainty on a PLQE value at 1 unit %.

Excitation scans were recorded on an Edinburgh Instruments FLS 980 Fluorimeter using an InGaAs detector for recording the IR emission (1300 ± 20 nm).

2.7 Transient Absorption

The short time (fs-ns) transient absorption setup has been described previously.[14] In summary, a Light Conversion PHAROS laser system with 400 µJ per pulse at 1030 nm with a repetition rate of 38 kHz was used. The output is divided, one part is focused onto a 4 mm YAG substrate to produce the continuum probe beam from 520 to 950 nm. The second part of the PHAROS output is lead into a narrow band optical parametric oscillator system (ORPHEUS-LYRA, Light conversion) outputting the pump beam. The probe pulse is delayed up to 2 ns with a mechanical delay-stage (Newport). A mechanical chopper (Thorlabs) is used to create an on-off pump-probe pulse series. The pump size on the sample is approximately 0.065 mm^2 and the probe about 0.015 mm^2. A silicon line scan camera (JAI SW-2000M-CL-80) fitted onto a visible spectrograph (Andor Solis, Shamrock) is used to record the transmitted probe light.

The longtime (ns-μs) transient absorption setup has also been described previously.[14] In short, the pump-probe setup consists of a probe from a LEUKOS Disco 1 UV supercontinuum laser (STM-1-UV, 1 kHz) and a pump generated in a TOPAS optical amplifier, pumped with the output from a Spectra-Physics Solstice Ace Ti:Sapphire amplifier (1 kHz). The probe beam is split into a reference and probe and both are focused onto the sample. A pair of line image sensors (Hamamatsu, G11608) mounted on a spectrograph (Andor Solis, Shamrock SR-303i) is used to detect the signal, using a custom-built board from Stresing Entwickslungsburo to read out the signal.

2.8 IR TCSPC

Samples were excited with a pulsed supercontinuum laser (Fianum Whitelase SC-400-4, 6 ps pulse length) at 0.2 MHz repetition rate. The pump wavelength set to either 535 nm or 650 nm (full-width at half-maximum 10 nm) with dielectric filters (Thorlabs). Pump scatter from the laser excitation within the photoluminescence path to the detector was filtered-out with an absorptive 900 nm long-pass filter (Thorlabs). The infrared photoluminescence was focused and detected by a single-photon avalanche photodiode based on InGaAs/InP (MPD-InGaAs-SPAD).

2.9 Steady-State and Magnetic Dependent PL

Similar to previously described temperature and current-controlled laser diodes (Thorlabs) were used to generate stable 532 nm and 658 nm laser beams.[14] The incident beam was attenuated as desired and focused onto the sample while PL from the sample was collected and focused into an Andor Kymera 328i Spectrometer and spectra recorded using either a Si-CCD (Andor iDus 420) for the visible region or an InGaAs detector (Andor, Dus InGaAs 490) for the NIR region. For magnetic field dependent PL measurements, an electromagnet was placed such that the sample was located within the poles of the electromagnet. As described previously,[15] the electromagnet was driven to achieve varying magnetic field strengths by using a Keithley 2400 variable voltage source connected to a current amplifier. The magnetic field between the poles (at the sample position) was calibrated to the applied voltage by a Gauss-meter. When measuring the PL from the PbS QDs (near IR region), both an RG1000 (Schott) and PL950 (Thorlabs) long pass filters were placed in front of the entrance to the spectrometer. These removed laser scatter and higher order peaks from the grating. After averaging over multiple sweeps of the magnetic field and integration of the spectra, the percentage change relative to the spectrum under zero applied field strength was calculated.

2.10 Synthesis of Ligands and Organic Host

Solvents were purchased in bulk from VWR, other reagents were purchased from Sigma Aldrich and were used as received. NMR spectra were measured on a 400 MHz or 600 MHz Bruker instrument, chemical shifts are reported in ppm and referenced to the deuterated solvents used. MALDI TOF MS was analyzed on a Bruker Microflex LRF with no matrix. X-ray diffraction data were collected at 90.0(2) K on either a Nonius kappaCCD diffractometer using MoK(alpha) X-rays or a Bruker-Nonius X8 Proteum diffractometer with graded-multilayer focussed CuK(alpha) X-rays. For the MoK(alpha) data, corrections for absorption

were applied using SADABS (Krause, L., Herbst-Irmer, R., Sheldrick, G.M. & Stalke, D. (2015). J. Appl. Cryst. 48, 3--10.). The structures were solved by direct methods (SHELXS (Sheldrick, G.M. (2008). Acta Cryst. A64, 112-122.)) and difference Fourier (SHELXL (Sheldrick, G.M. (2015). Acta Cryst. C71, 3--8.)). Refinement was carried out against F^2 by weighted full-matrix least-squares (SHELXL). Hydrogen atoms were found in difference maps but subsequently placed at calculated positions and refined using riding models. Non-hydrogen atoms were refined with anisotropic displacement parameters. Atomic scattering factors were taken from the International Tables for Crystallography (International Tables for Crystallography, vol C: Mathematical, Physical and Chemical Tables. A.J.C. Wilson, Ed. (1992). Kluwer Academic Publishers, Holland.).

We initially attempted to prepare the carboxylic acid ligand following the same approach used to prepare TIPS Tetracene carboxylic acid[16] – beginning with an appropriately brominated quinone, a lithium acetylide is added to the carbonyl moieties to form an intermediate dialkoxide. This addition is immediately followed by in-situ metal/halogen exchange and carboxylation, and finally a deoxygenative workup (Scheme 1). This route seemed particularly appealing, because the requisite bromoquinone (Q-Br) was reported to be easily prepared.[17] After following the procedure shown in Scheme 1 (top), the isolated product did not yield the expected NMR spectrum, so crystals were grown. X-ray analysis of these crystals did not yield a well-refined structure, but it did suggest the presence of substantial substitution of bromine on the 3-position of the thiophene, along with carboxylic acid on the 2-position. To determine whether this product arose from an unusual ‘halogen dance reaction,[18] or whether the starting material had been mis-identified, we prepared the corresponding trimethylsilylethynyl derivative in good yield (Scheme 1, middle), which yielded excellent crystals. The well-resolved structure shows the bromine substituent strictly on the 3-position of the chromophore, suggesting that the structure of the product from the literature synthesis was incorrect (no crystallographic analysis was reported in that work). It is thus likely that structures from that, and other publications using the same method,[19] likely do not possess the assigned structures, since none have been confirmed crystallographically, and the difference in ^{1}H NMR spectra will be subtle.

We recently demonstrated that thienoacenes are very amenable to simple deprotonation and carboxylation using LDA. Starting from known triisopropylsilylethynyl substituted thienoanthracene,[20] were able to make the corresponding carboxylic acid in 52% isolated yield (see below).

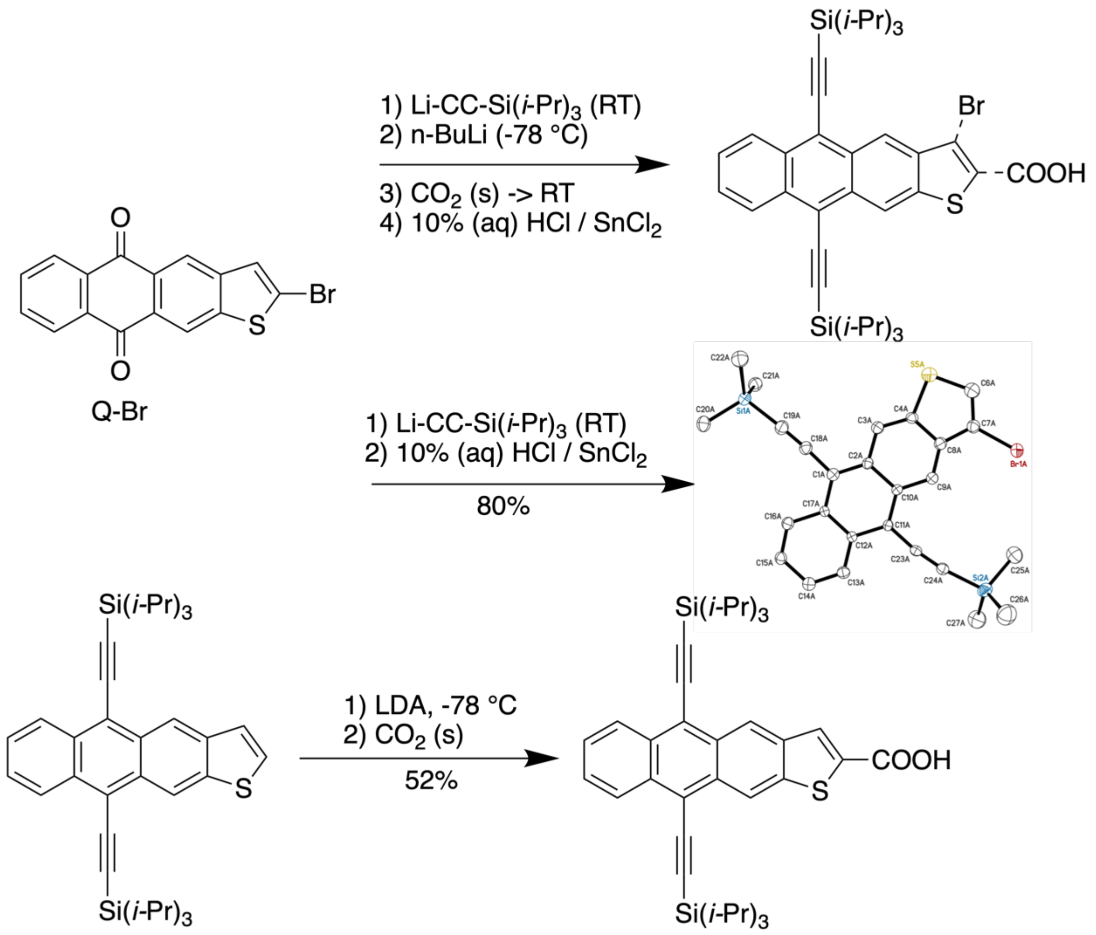

Scheme 1. *Preparation of TA-CA.*

3-bromo-5,10-bis(trimethylsilylethynyl) thienoanthracene: To a solution of trimethylsilyl acetylene (1.27 mL, 9 mmol) in 24 mL anhydrous THF in a flame-dried flask with stir bar that had been flame dried and cooled under nitrogen was slowly added 3.4 mL of a 2.5 M solution of n-BuLi in hexanes (8.5 mmol). After stirring at room temperature for 30 minutes, 1 gram of purported “2”-bromo thienoanthracene 5,10-quinone (prepared as described in reference 17) was added in one portion, and the mixture allowed to stir until all quinone had dissolved. Three hours after complete dissolution, the reaction mixture was quenched with 5 mL of 10% aqueous HCl, followed by 6 g (27 mmol) of stannous chloride dihydrate. This mixture was allowed to stir for 30 minutes, and was then poured into hexanes and extracted three times with water. The organic phase was then dried over magnesium sulfate, and poured directly through a thin (2 cm) silica gel plug (hexanes). After removal of solvent, the resulting solid was recrystallized from acetone to yield 1.2 g (80%) of the 3-bromo thienoanthracene, as confirmed by crystallographic analysis. ^{1}H NMR (400 MHz; $CDCl_3$) δ 9.10—9.08 (m, 2H), 8.61—8.55 (m, 2H), 7.61—7.57 (m, 3H), 0.48 (d, J = 8.4 Hz, 18H). ^{13}C NMR (101 MHz; $CDCl_3$): δ 137.65, 137.65, 137.49, 137.49, 132.69, 132.69, 132.06, 132.06, 130.03, 130.03, 127.57, 127.57, 127.52, 127.52, 127.36, 127.36, 127.24, 127.24, 126.94, 126.94, 126.88, 126.88, 121.81, 121.81, 120.89, 120.89, 119.32, 119.32, 117.49, 117.49, 109.66, 109.66, 108.91, 108.91, 107.87, 107.87, 101.75, 101.75, 77.16, 77.16, 0.39, 0.39, 0.37, 0.37. HRMS (LDI): Calculated: 504.0399; Found: 504.0404.

5,10-bis(triisopropylsilylethynyl) thienoanthracene-2-carboxylic acid (TA-CA): To a flame-dried flask with stir bar that had been cooled under a constant flow of dry nitrogen, 5,10-bis(triisopropylsilylethynyl)

thienoanthracene (prepared as described in reference 20, 1.19 g, 2 mmol) was dissolved in 5 mL anhydrous THF. The reaction mixture was cooled to -78 °C and 10 mL of 1M LDA (5 eq) were added dropwise, and the reaction mixture was stirred for approximately 45 minutes at that temperature. Solid CO_2 was next added in great excess, and the reaction mixture was stirred for an additional 20 minutes. The reaction was quenched with 10% HCl and extracted with ethyl acetate. The organic layers were combined, dried with magnesium sulfate, and concentrated via rotary evaporation. The product was purified on silica with ethyl acetate and recrystallized with acetone to give small red crystals (0.66 g, 52%. ^{1}H NMR (400 MHz, D_8THF): δ 9.389 (d, J = 0.95 Hz, 1H), 9.374 (s, 1H), 8.807 (m, 2H), 8.159 (s, 1H), 7.352 (s, 2H), 1.304 (d, J = 4.37 Hz, 42H). HRMS (LDI): Calculated: 638.3070; Found: 638.3081.

((2-fluoroanthra[2,3-b]thiophene-5,10-diyl)bis(ethyne-2,1-diyl))bis(triisobutyl-silane (F-TA):

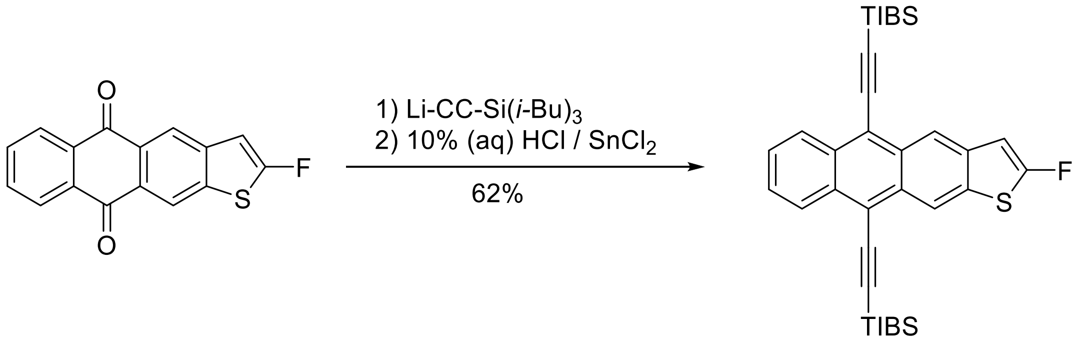

Scheme 2. *Preparation of F-TA*

2-fluoro thienoanthracene quinone[21] and triisobutylsilyl acetylene[22] were prepared by the previously reported literature methods.

In a flame dried 100 mL round bottom flask, 1.11 g of tri(isobutyl)silyl acetylene (5.05 mmol) was dissolved in 10 mL of n-heptane and cooled to 0 °C. 1.96 mL of 2.5 M n-BuLi (4.89 mmol) was added dropwise to the reaction solution and stirred for 30 minutes. The reaction solution was diluted with 40 mL of n-heptane. 0.36 g of 2-fluoro-5,10-thienoanthracene quinone (1.27 mmol) was added to the reaction solution, followed by 7 mL of dry THF. The reaction mixture was brought to room temperature and stirred for 16 hours. The reaction was quenched with water, extracted with DCM and the combined organic phases washed with water. The organic phase was dried over $MgSO_4$, filtered, and the solvent evaporated in vacuo. The resulting yellow oil was run through a thick pad of silica using HPLC-grade hexane as eluent to remove excess acetylene, followed by dichloromethane to collect the intermediate product, ethynylated thienoantracene diol as a yellow oil which was immediately used in the next step. The ethynylated thienoanthracene diol was dissolved in 15 mL of THF and 15 mL of 10% HCl, 1.41 g of $SnCl_2 \cdot 2H_2O$ (5.64 mmol) was added to the reaction mixture along with 20 mL of THF and stirred for 1 hour. The solution was extracted with the HPLC-grade hexane and dried over $MgSO_4$, the solvent evaporated in vacuo. The resulting orange solid was purified over a thick silica plug, using HPLC-grade hexane as eluent. After the removal of solvent, the final product, F-TA was collected as a yellow solid (0.38 g, 62%. ^{1}H NMR (600 MHz, $CDCl_3$) δ 8.95 (s, 1H), 8.86 (s, 1H), 8.64 – 8.47 (m, 2H), 7.67 – 7.46 (m, 2H), 6.77 (d, *J* = 2.1 Hz, 1H), 2.14 – 2.04 (m, 6H), 1.11 (d, *J* =

6.6 Hz, 36H), 0.88 (d, *J* = 6.9 Hz, 12H). ^{13}C NMR (101 MHz, $CDCl_3$): δ 167.66, 164.71, 137.12, 137.05, 134.40, 132.99, 132.68, 131.15, 130.67, 130.64, 127.67, 127.59, 127.15, 127.10, 121.42, 121.33, 121.01, 118.91, 118.33, 108.39, 108.26, 104.56, 104.38, 102.96, 102.85, 26.713, 26.69, 25.73, 25.65. ^{9}F NMR (376 MHz, C_6D_6): δ 117.55. M.S. (EI, 70 eV); 696 (15%, M^+), 639 (100%, M^+ – i-Bu). HRMS (LD): expected: 696.4017 found: 696.4000.

2.11 Photon Upconversion Measurements

2.11.1 Upconversion in Solution

Following the De Mello method[23] The solution upconversion measurements were determined in an integrating sphere fiber-coupled to a spectrograph (Shamrock SR-303i, ANDOR) with CCD camera (Andor iDus DU420A Si CCD, ANDOR), calibrated for spectral sensitivity of the detector at each wavelength. The upconverted emission was corrected for reabsorption using a spectra obtained from a solution with low concentration of the annihilator.

2.11.2 Upconversion in Films

Absorption spectra were measured of the films using a spectrometer (UV3600-Plus, Shimadzu) equipped with an integrating sphere. Upconverted emission spectra was measured using a spectrograph (Shamrock SR-303i, ANDOR) with CCD camera (Andor iDus DU420A Si CCD, ANDOR), calibrated for spectral sensitivity of the detector at each wavelength. Two CW lasers of 790 nm and 405 nm (Thorlabs) were overlapped on the sample from the side facing the detector. The emission was then recorded with either the 790 nm or 405 nm laser blocked to record the reference and upconverted spectra, respectively. The emitted light passed through a 750 nm short pass filter (Thorlabs) before the detector. The intensity was recorded with a power meter (Thorlabs) and the spot size at the sample was determined using a beam profiler (Thorlabs). The quantum yield under 405 nm excitation was first determined for the films using an integrating sphere (described above) following the De Mello method.[23] The photon upconversion quantum yield (Φ_{UC}) of the film was determined using the relative method using the relationship:

$$\Phi_{UC} = \Phi_{405}\left(\frac{1-10^{-A_{405}}}{1-10^{-A_{790}}}\right)\left(\frac{F_{790}}{F_{405}}\right)\left(\frac{I_{Ex,405}}{I_{Ex,790}}\right) \quad \text{(S8)}$$

Where Φ_{405} is the quantum yield of fluorescence when exciting the films with 405 nm light, A_i is the absorption at excitation wavelength *i*, and F_i is the integrated emission intensity. $I_{Ex,i}$ is the excitation photon flux at wavelength *i.*

Intensity dependence measurements where recorded in a similar manner attenuating the 790 nm excitation light using a graduated ND-filter.

3 TEM of SF-PM films

Figures S2 and S3 show TEM images of PbS-OA:TIPS-Tc and PbS-TET-CA:TIPS-Tc films. The PbS-OA and PbS-TET-CA QDs displayed similar size distributions as imaged within TIPS-Tc films. PbS-OA showed

significant aggregation whilst PbS-TET-CA showed well-dispersed QDs despite high QD loadings. The more favourable interaction of TET-CA ligands with the TIPS-Tc compared to oleic acid prevented the phase segregation behaviour observed in the PbS-OA films.

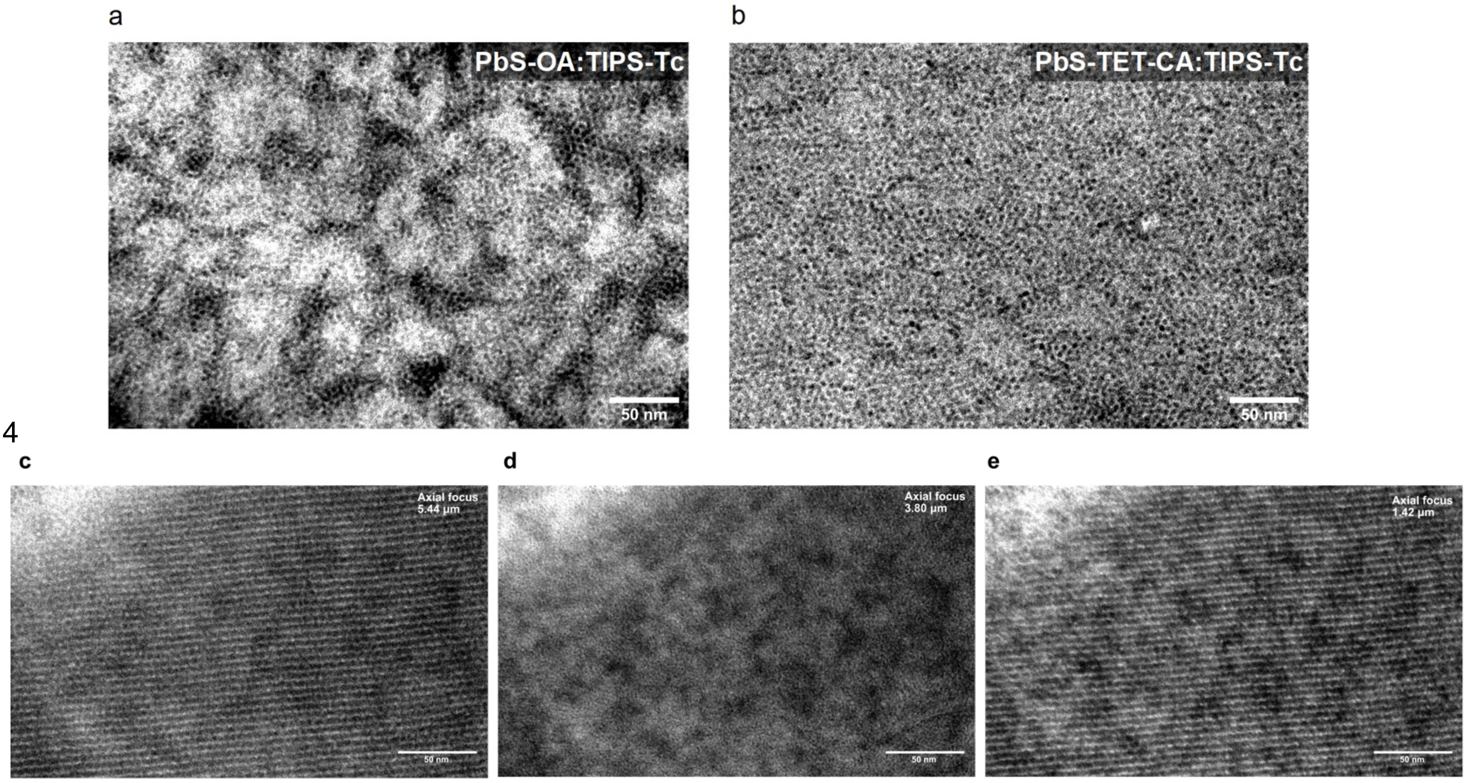

Supplementary Figure 4*: TEM at the 50 nm scale, a) PbS-OA:TIPS-Tc b) PbS-TET-CA:TIPS-Tc films. The PbS-OA:TIPS-Tc films show clear phase separation, with large aggregates of PbS-OA QDs (dark dots) within the SF-host (lighter regions). While the PbS-TET-CA:TIPS-Tc films are significantly more homogeneously dispersed throughout the TIPS-Tc SF-host material. c-e) PbS-TET-CA:TIPS-Tc film at different focus depth: c) 5.44 μm, d) 3.80 μm, e) 1.42 μm.*

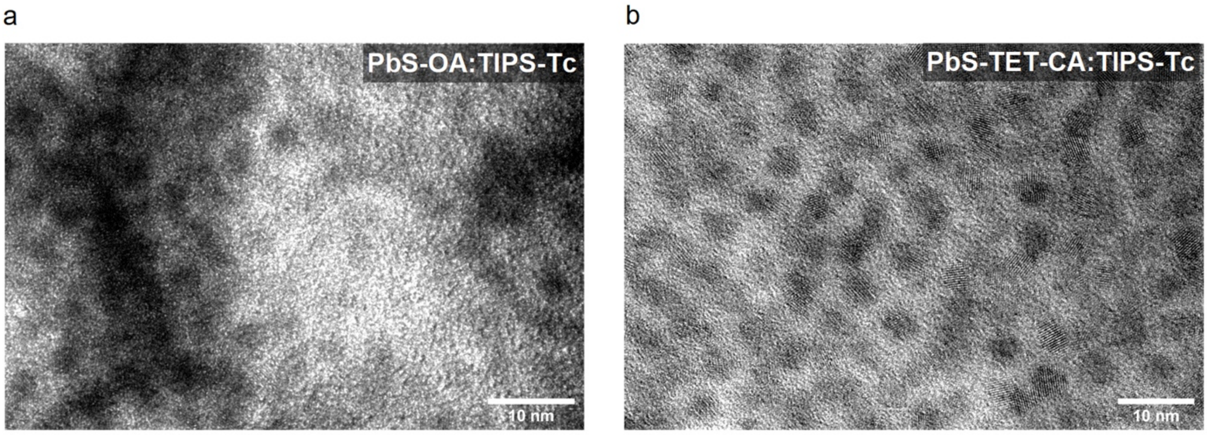

***Supplementary Figure** 5: TEM at the 10 nm scale, a) PbS-OA:TIPS-Tc b) PbS-TET-CA:TIPS-Tc films. In both films, we observe interference patterns inside the QDs (dark dots) assigned to the PbS crystal packing.*

Table S3: *PbS QDs diameter values and standard deviation as measured from the TEM images of PbS-OA:TIPS-Tc and PbS-TET-CA:TIPS-Tc films.*

Sample (50 mg QD:100 mg TIPS)	QD Mean (nm)	Standard deviation (nm)
PbS-OA	3.9	0.4
PbS-TET-CA	3.9	0.3

4 Extracting an Exciton Multiplication Factor for SF-PM films

Here, the focus is on the TIPS-Tc triplet exciton photo-physics in the TIPS-Tc, thus the following description neglects the effects of a possible intermediate state facilitating the transfer between the SF-host and QD. The overall SF-PM efficiency is given by,[24]

$$\frac{\eta_{PM}(\lambda)}{\eta_{QD}} = \alpha_{QD}(\lambda) + \alpha_{TIPS-Tc}(\lambda)\eta_{EMF} \quad \text{(S9)}$$

Where η_{PM} and η_{QD} are the IR photoluminescence quantum efficiencies (PLQE) when exciting the SF material and QD, respectively. α_i is the fractional absorption of component i. To apply this model for the triplet harvesting the IR PLQEs under 515 nm and 658 nm excitation were measured.[14] Due to the disordered and polycrystalline nature of the TIPS-Tc films, we assume the fractional absorption of each component, α_i, are well approximated by the previously measured values in the solution phase, ***Supplementary Figure 6***.[14] We calculate the exciton multiplication factor η_{EMF} by rearranging the equation above for η_{EMF}. For the PbS-TET-CA:TIPS-Tc film we measure values of η_{QD} = (15.4 ± 1.0) % (excitation at 658 nm, QD only) and $\eta_{PM}(\lambda = 515\,nm)$ = (24.5 ± 1.0) % (excitation at 515 nm, QD + SF host). Taking the values for $\alpha_{QD}(\lambda)$ = 0.31 and $\alpha_{TIPS-Tc}(\lambda)$ = 0.69 at 515 nm and calculating the propagation of uncertainties, leads to η_{EMF} = (186 ± 18) %.[14] The measurement under the TIPS-Tc triplet decay rate by ns-TA gives us a triplet harvesting efficiency of η_{TET} = (97 ± 11) % (section 7). Using $\eta_{EMF} = \eta_{SF}\eta_{TET}$ we find a singlet fission yield of η_{SF} = (192 ± 28) %.

In contrast, for the PbS-OA:TIPS-Tc film we measure values of η_{QD} = (17.2 ± 1.0) % and $\eta_{PM}(\lambda = 515\,nm)$ = (3.8 ± 1.0) %. Taking the values for $\alpha_{QD}(\lambda)$ = 0.25 and $\alpha_{TIPS-Tc}(\lambda)$ = 0.75 at 515 nm and calculating the propagation of uncertainties, leads to η_{EMF} = (-4 ± 8) %.[14] Which indicates that there is essentially no triplet harvesting occurring in the PbS-OA:TIPS-Tc film.

5 Absorbance Spectra of SF-PM film

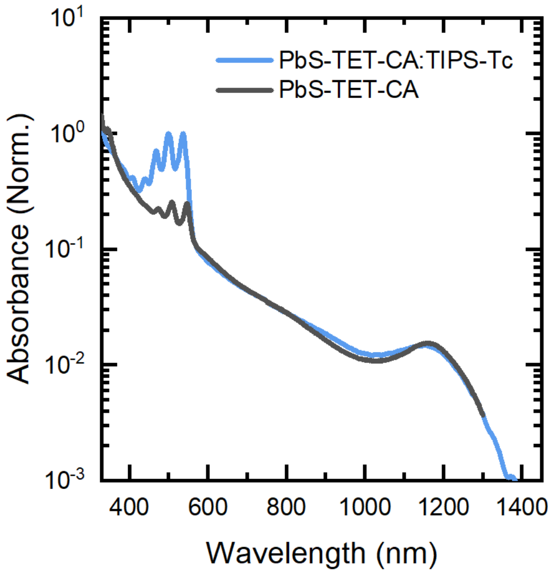

***Supplementary Figure** 6. Normalised absorbance spectra of a PbS-TET-CA:TIPS-Tc film (blue) and a solution of PbS-TET-CA QDs in toluene.*

6 Magnetic Field Dependent PL of SF-PM films

Direct excitation of the QD in PbS-TET-CA:TIPS-Tc films with 658 nm laser light results in no observed magnetic dependence (for fields less than 0.5 T), similar to previous observations.[11, 12, 14] Under 532 nm excitation the TIPS-Tc singlet emission shows an increased PL on the application of high magnetic fields (>0.3 T), as expected for a singlet state undergoing singlet fission (Figure 3c in main text). While, the PbS-TET-CA IR PL shows a corresponding decrease, indicating that the excited QD states are the result of triplets generated by singlet fission, transferred from the TIPS-Tc.[14,15,25]

7 Picosecond Transient Absorption of SF-PM films

ps-Transient absorption (TA) was done on TIPS-Tc films with and without PbS-TET-CA quantum dots, *Supplementary Figure 7*. The kinetics and spectral evolution of TIPS-Tc features are very similar in both cases, indicating no effect of the presence of quantum dots on the SF dynamics. The small difference is mainly due to the underlying distortion of the quantum dot dynamics which inevitably are excited at the same wavelength (535 nm).

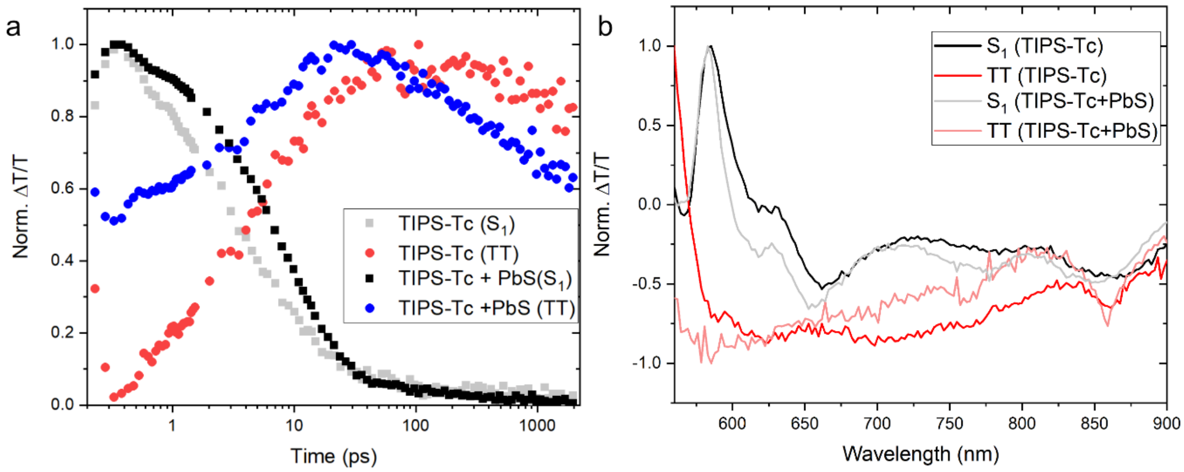

***Supplementary Figure 7**: a) Normalised transient absorption kinetics and b) spectral shape, comparing TIPS-Tc films with PbS-TET-CA:TIPS-Tc films. The kinetics in a) is the raw data extracted at 580 nm (S1) and 870 nm (TT) where the singlet and TT features are dominant. Therefore, any overlapping quantum dot signal will affect the kinetic trace. Still, only small changes, mainly explained by the overlapping quantum dot features, indicate that the singlet-fission process is unperturbed by the addition of quantum dots.*

8 Nanosecond Transient Absorption and IR Transient PL of SF-PM films

To further characterise the PL enhancement of the SF-PM films and gain a mechanistic understanding of the triplet transfer to the QD, we used ns-resolved transient absorption (ns-TA) and near-infrared (NIR) time-correlated single photon counting (TCSPC).

8.1 Nanosecond Transient Absorption (ns-TA)

The spectrally narrow TIPS-Tc triplet photoinduced absorption (PIA) features at 850 and 970 nm readily lend themselves to the extraction of the triplet population dynamics in the blend films (*Supplementary Figure 8-11*). The PIA kinetics (965-980 nm) in pristine TIPS-Tc films and PbS-TET-CA:TIPS-Tc films were fitted to extract the intrinsic triplet decay rate k_1 and the triplet transfer rate k_{TET1}. A comparison of the TIPS-Tc triplet lifetime in films of TIPS-Tc, PbS-OA:TIPS-Tc and PbS-TET-CA:TIPS-Tc is shown in *Supplementary Figure 8*. Based on the increase in monomolecular TIPS-Tc triplet decay rate a significant triplet quenching of (97 ± 11) % is estimated for the film containing PbS-TET-CA quantum dots. Assuming that this triplet quenching is caused by triplet transfer to the QDs we estimate the singlet fission yield to be η_{SF} = (192 ± 28) %, based on η_{TET} = (97 ± 11) % and the exciton multiplication factor η_{EMF} = (186 ± 18) % calculated above. The high SF yield is in line with previous reports and indicates that the process is not influenced by the doping of TIPS-Tc with PbS-TET-CA QDs (see also *Supplementary Figure 7*).[26]

Supplementary Figures 8-9 show in the measured nanosecond TA of TIPS-Tc, PbS-TET-CA:TIPS-Tc and PbS-OA:TIPS-Tc films, we observe TIPS-Tc triplet photo-induced absorption features (PIA) at ~860 and ~970 nm, after excitation of the TIPS-Tc (515 nm). These triplet PIA features are significantly quenched in the PbS-TET-CA:TIPS-Tc films, an indication of triplet transfer (Supplementary Figure 11). Fitting of the triplet PIA kinetics at ~970 nm with mono-exponential decays allows the extraction of the triplet intrinsic decay rate and the triplet transfer rate (Table S4). For PbS-TET-CA:TIPS-Tc films we extraction a triplet transfer rate of 0.34 ± 0.03 μs^{-1}, which corresponds to a triplet transfer efficiency of (97 ± 11) %.

Due to the overlapping absorption of the TIPS-Tc and PbS QD absorption excitation with 515 nm pump leads to excitation of the QD directly, as observed by the positive nsTA feature assigned to QD ground state bleach (GSB) at ~1150 nm (Supplementary Figures 8 and 9). A further consequence of this low absorption contrast is that the comparison of the QD GSB decay under 515 and 650 nm excitation does not show a significant difference in lifetime, Supplementary Figure 10. As an alternative, transient IR PL is used to gain insight into the transfer of triplet exciton to the PbS QDs (See section 8.4 and 8.5). Supplementary Figure 11 shows fitting of the QD GSB kinetics with mono-exponential decays suggesting 72 ± 2 ns and 306 ± 14 ns lifetimes of the PbS-OA and PbS-TET-CA QDs in the PbS:TIPS-Tc films (intrinsic response, 650 nm excitation).

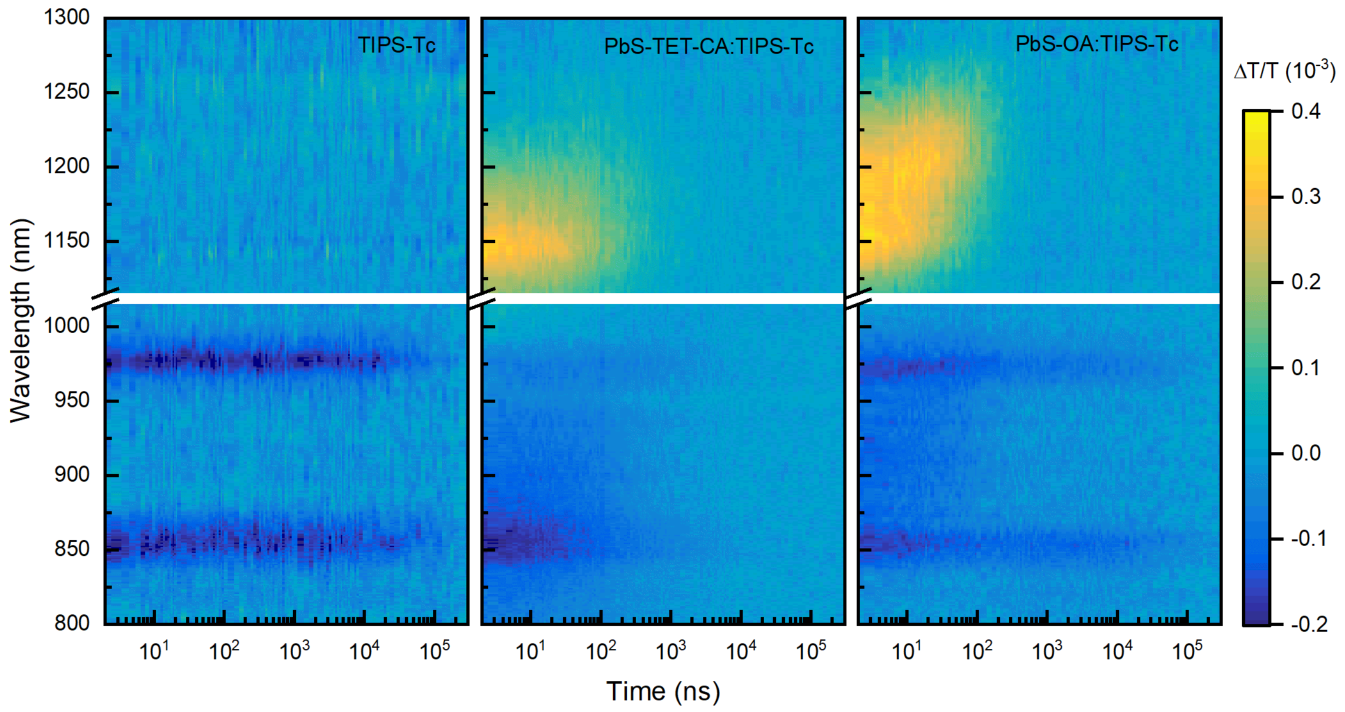

Supplementary Figure 8. Nanosecond transient absorption maps for films of TIPS-Tc, either pristine, with PbS-TET-CA or PbS-OA QDs, excited at 515 nm with ~15 μJ/cm^2. The TIPS-Tc triplet PIA peaks at 850 and 970 nm are clear in all cases. However, the triplet lifetime varies between the films, the PbS-Tet_CA:TIPS-Tc film having the shortest lifetime. Predominately due to direct photoexcitation, in the SF-PM systems the QD GSB is observed at 1100-1250 nm from early times (<2 ns). In the PbS-OA:TIPS-Tc there is clear red-shifting of the QD GSB within the first 10 ns after photoexcitation.

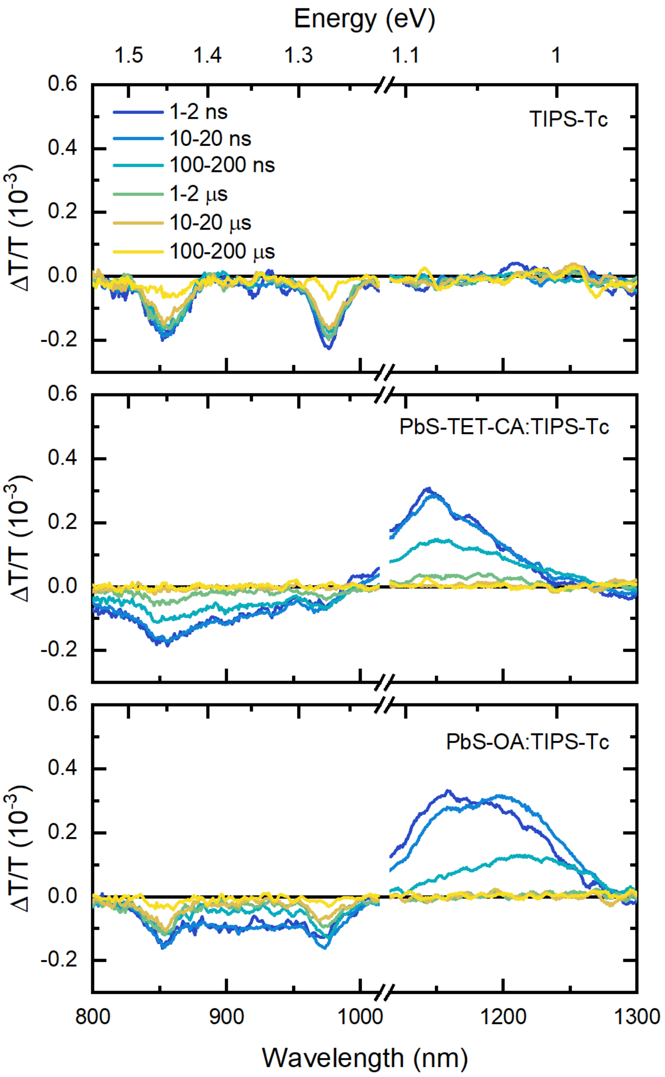

Supplementary Figure 9. Nanosecond transient absorption spectra for films of TIPS-Tc, either pristine, with PbS-TET-CA or PbS-OA QDs, excited at 515 nm with ~15 μJ/cm^2. Transient absorption spectra are averaged over the time ranges indicated. The TIPS-Tc triplet PIA peaks at 850 and 970 nm are clear in all films. Predominately due to direct photoexcitation, in the SF-PM systems the QD GSB is observed at 1100-1250 nm from early times (<2 ns). In the PbS-OA:TIPS-Tc there is clear red-shifting of the QD GSB within the first 10 ns after photoexcitation.

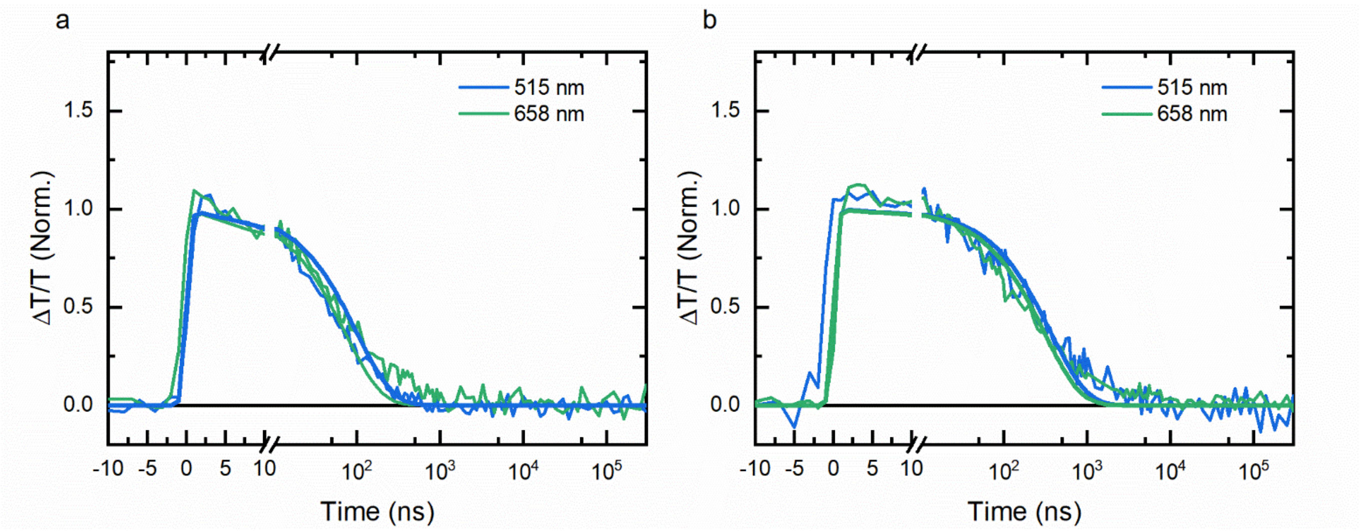

Supplementary Figure 10. Nanosecond transient absorption PbS QD GSB kinetics (1120-1180 nm) for films of PbS-OA:TIPS-Tc (a) and PbS-TET-CA:TIPS-Tc (b), excited at either 515 or 658 nm with ~15 μJ/cm^2. The kinetics have been fit with mono-exponential decays. The PbS-OA GSB decays with a 100 ± 6 ns and 72 ± 2 ns lifetimes when excited at 515 and 658 nm respectively. The PbS-TET-CA GSB decays with a 370 ± 25 ns and 306 ± 14 ns lifetimes when excited at 515 and 658 nm respectively.

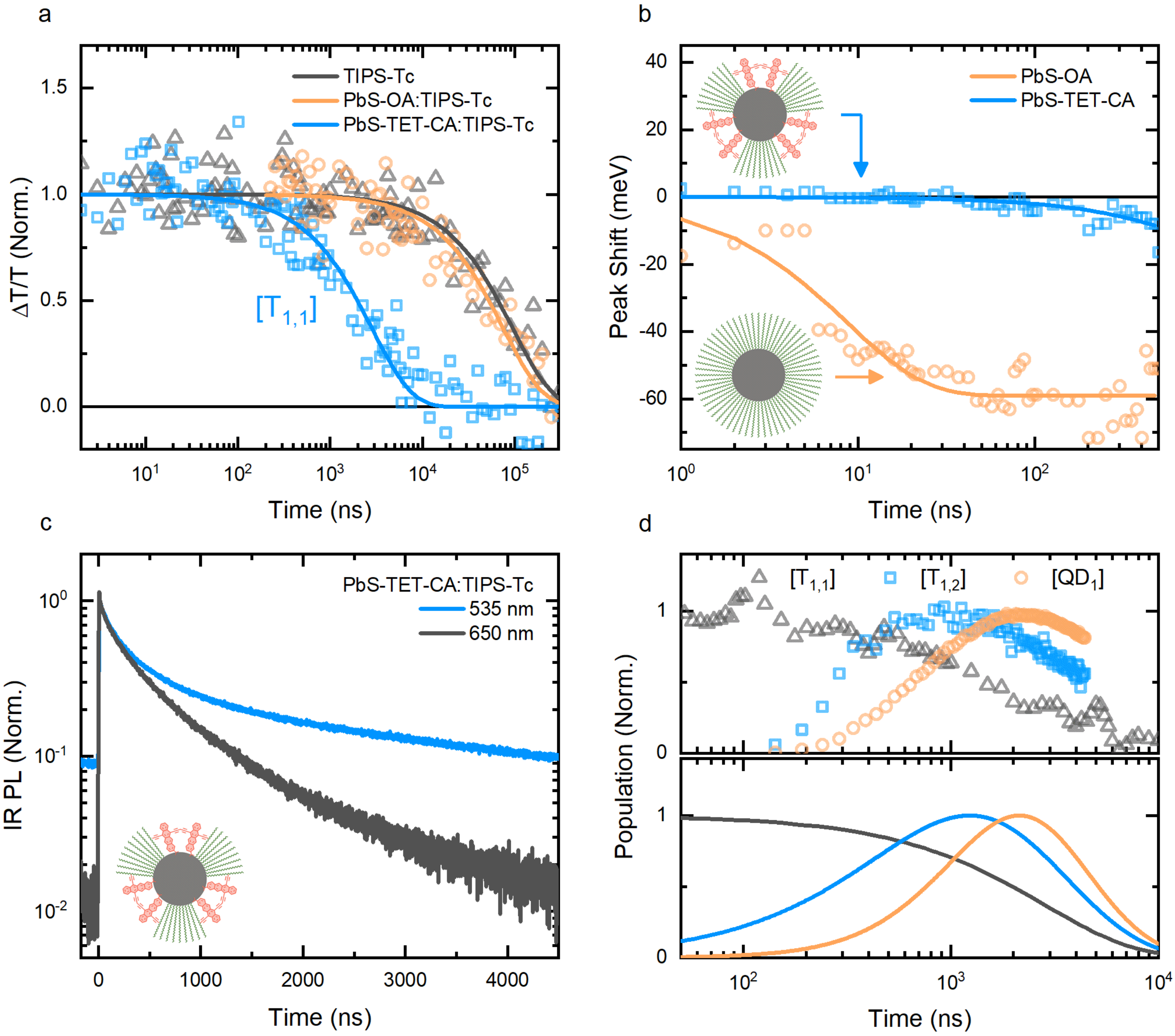

Supplementary Figure 11. (a) Normalised transient absorption kinetics at the TIPS-Tc triplet PIA (965-980 nm) for films of TIPS-Tc, either pristine (light grey triangles), with PbS-OA (light orange circles) or with PbS-TET-CA quantum dots (light blue squares), excited at 515 nm with ~15 µJ/cm^2, with mono-exponential fits (darker curves).(**b)** The shift in the peak position of the PbS quantum dot's GSB, in films of PbS-OA:TIPS-Tc (light orange circles) and PbS-TET-CA:TIPS-Tc (light blue squares) after excitation with 658 nm at ~15 µJ/cm^2, with mono-exponential fits (darker curves). **(c)** Normalised transient IR PL for a film of PbS-TET-CA:TIPS-Tc under 535 nm (blue curve) and 650 nm excitation (black curve), at ~0.015 and 0.010 µJ/cm^2 respectively. **(d)** The decay of the TIPS-Tc triplets [$T_{1,1}$] (black) transferring to TET-CA triplets [$T_{1,2}$] (blue) and then finally leading to emission from the excited PbS quantum dot state [QD_1] (orange). **d, top)** [$T_{1,1}$] determined from nsTA, while the [$T_{1,2}$] and [QD_1] populations were determined from transient IR PL. **d, bottom)** Associated fits to the dynamics in the three-state kinetic scheme.

8.2 Analysis of the QD GSB Shifting

As shown in Supplementary Figures 8 and 9, it is apparent that under direct excitation (650 nm) the peak of the ground-state bleach (GSB) signal for the PbS-OA QDs in the blend shifts to lower energies by roughly 60 meV within 20 ns, whereas the PbS-TET-CA QD in the blend have a less than 10 meV shift over their entire excited state lifetime. We monitor the QD GSB to track the relaxation of QD excited state population to low-energy sites. As the excited states transfer to lower energy QD sites the GSB in nsTA is red-shifted. Extraction of the peak position of the GSB allows tracking of the excited state relaxation (Supplementary Figure 12). We convert the wavelength shift to an energy change and fit the shift in energy to a mono-exponential decay. The PbS-OA QDs show a dramatically faster and larger drop in the energy of the QD excited states, indicating a higher degree of QD to QD transfer associated with aggregation.

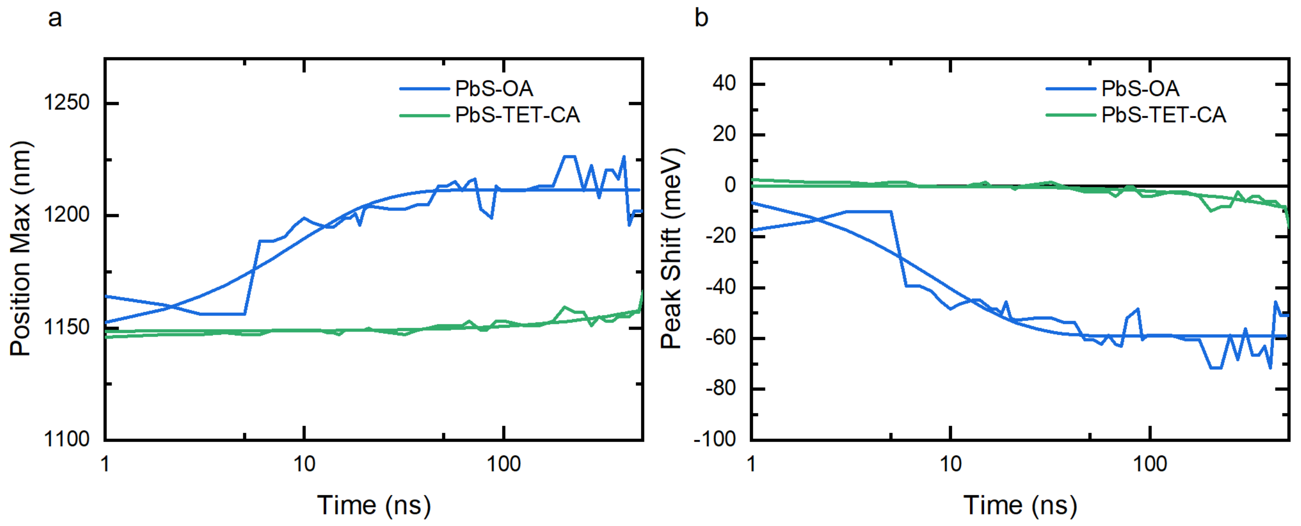

Supplementary Figure 12: Wavelength of the maximum signal for the GSB of PbS QD in films of PbS-OA:TIPS-Tc and PbS-TET-CA:TIPS-Tc after excitation with 658 nm at ~15 µJ/cm^2. a) peak position (b) and relative peak position shift for the PbS-OA and PbS-TET-CA GSB after 658 nm pump excitation. The shifts in peak wavelength have been parameterised with an exponential decay with offset. The peaks of the PbS-

OA QD GSB drops by 60 ± 10 meV, with a time constant of 9 ± 1 ns. While the peaks of the PbS-TET-CA QD GSB drops by 10 ± 5 meV, with a time constant of 1500 ± 200 ns.

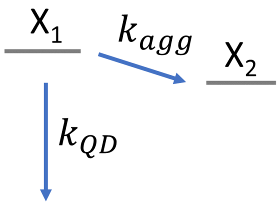

***Supplementary Figure 13**: Competition between exciton decay and transfer to low energy QD sites. Illustration off the simple kinetic model used to capture the branching between aggregation assisted hoping to lower QD sites and isolated QD decay rate.*

We construct a kinetic model that assumes a simplistic branching between QD excited state decay and transfer to a low energy QD site (Supplementary Figure 13). Within this model, we estimate the QD aggregation-assisted trapping efficiency as,

$$\eta_{agg} = \frac{k_{agg}}{k_{agg}+k_{QD}} \;. \qquad (S10)$$

Taking the rates from the fitting to the position of the QD GSB peak and the decay of the total area under the GSB we calculate assisted trapping efficiencies of (90 ± 20) % and (17 ± 2)% for the OA and TET-CA capped QDs respectively.

8.3 Kinetic Parameters Extracted from nsTA

***Table S4:** Kinetic parameters obtained from fitting nanosecond transient absorption kinetics. Triplet intrinsic and transfer rates are calculated from fitting of mono-exponential functions to the TIPS-Tc triplet PIA at 865-980 nm, under 515 nm excitation at ~15 μJ/cm². While the QD intrinsic decay rate is established from a mono-exponential fit to the GSB at 1120-1180 nm, under 658 nm excitation at ~15 μJ/cm². The triplet exciton transfer efficiency, η_{TET}, is calculated from the ratio between the triplet transfer rate and the sum of all relevant triplet decay channels.*

	k_1 (1/us)	k_{TET} (1/us)	η_{TET} (%)	k_{QD} (1/us)	k_{agg} (1/us)	η_{agg} (%)
Measurement	TA fit	TA fit	TA fit	TA fit	TA fit	TA fit
TIPS-Tc	(1.05 ± 0.10)x10-2	-	-	-	-	-
PbS-TET-CA:TIPS-Tc	(1.05 ± 0.10)x10-2	0.34 ± 0.03	0.97 ± 0.11	3.27 ± 0.15	0.68 ± 0.08	17 ± 2
PbS-OA:TIPS-Tc	(1.05 ± 0.10)x10-2	0.0028 ±0.0016	0.20 ± 0.15	13.8 ± 0.4	110 ± 20	90 ± 20

8.4 IR Transient PL (IR TCSPC)

Time-correlated single photon counting was employed to measure the IR transient PL under excitation of the QDs (650 nm) and the SF host (535 nm) in both PbS-TET-CA:TIPS-Tc and PbS-OA:TIPS-Tc films

(Supplementary Figure 11c-d and Supplementary Figures 15-17). We measure an instrument response function (IRF) with a full-width at half maximum of 5.5 ± 0.5 ns for the IR transient PL setup (Supplementary Figure 14). This IRF is shorter by 2 orders of magnitude than any of the time constants we observe in the triplet transfer processes. As such we treat the IRF as instantaneous relative to the dominant triplet transfer processes.

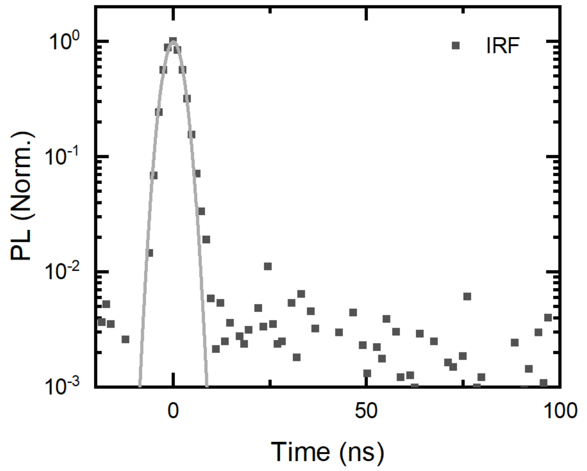

***Supplementary Figure 14**. IR TCSPC instrument response function (IRF). Collected using a scattering glass substrate with 650 nm laser scatter. Fitting the IRF with a Gaussian peak function is obtained with a full-width at half maximum of 5.5 ± 0.5 ns.*

With the laser excitation blocked before entering the sample area, the detector counts due to ambient conditions were measured for the same exposure time as the transient PL measurements. The mean detector counts per time bin, across the time window, represents the background PL counts. The PL kinetics were correct by subtraction of this value. After subtraction of this background value, positive values for the PL kinetics at times before the laser pulse are observed. These PL levels ($t < 0$) arise due to the periodic nature of the experiment. Periodicity in the PL kinetics is explicitly included in the time-series deconvolution analysis (see below) due to the periodicity of the fast Fourier transform. We perform fluence-dependent measurements to investigate the effect of any non-linear behaviour of the QD excited state (650 nm excitation) or TIPS-Tc triplet decay (535 nm excitation) (Supplementary Figure 15). Over the range of incident fluences investigated, we observed no dependence of the transient IR PL decay, indicating even at the highest fluence used 15 nJ/cm^2 the system is in the low excitation density regime where bimolecular decay in the QD or the TIPS-Tc triplet can be ignored. While the IR PL shows an extended lifetime under excitation of the TIPS-Tc (535 nm) relative to excitation of the QD alone (650 nm) in the PbS-TET-CA:TIPS-Tc film (Supplementary Figure 11 and 15), the PbS-OA:TIPS-Tc does not show any significant extension (Supplementary Figure 16), in agreement with the steady-state observation that effectively no triplet transfer is occurring. We extract QD excited state decay rates of 2.3 ± 0.2 μs^{-1} and 2.5 ± 0.2 μs^{-1} by fitting the transient IR PL decay of PbS-OA:TIPS-Tc and PbS-TET-CA:TIPS-Tc respectively, under 650 nm excitation (Supplementary Figure 17).

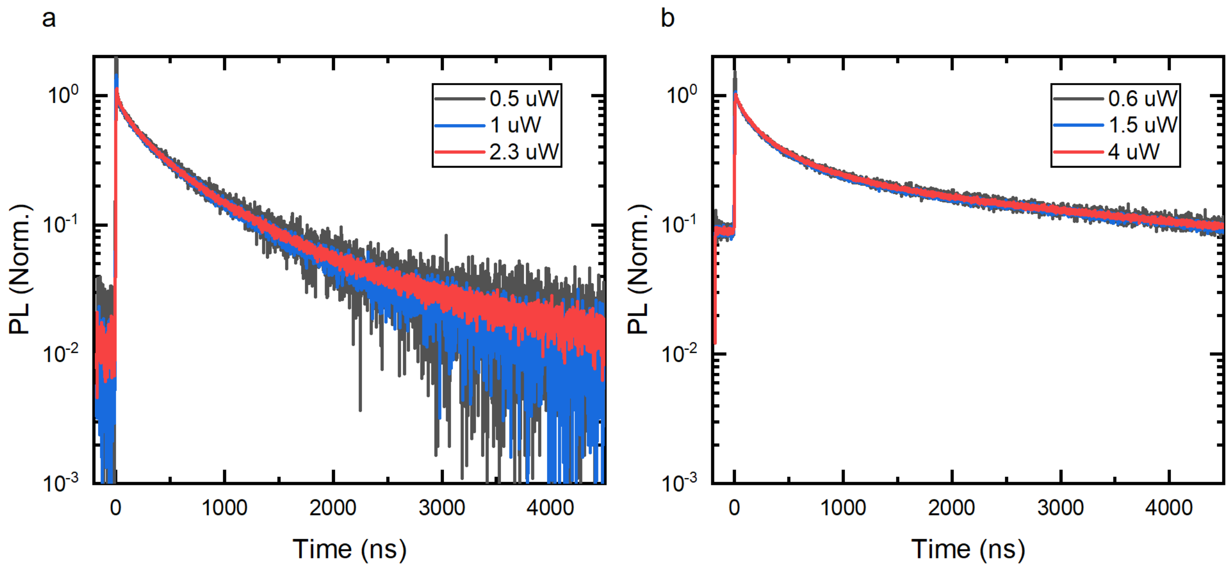

Supplementary Figure 15. *Normalised IR transient PL kinetics for a PbS-TET-CA:TIPS-Tc film. The PbS-TET-CA:TIPS-Tc film was excited at 650 nm (a) and 535 nm (b) with varying fluences. 650 nm excitation at ~2.5, 5, 10 nJ/cm² was used. 535 nm excitation at ~2, 7 and 15 nJ/cm², at 0.2 MHz repetition rate was used. Contribution to the detected counts by background counts was removed before normalisation to the initial value of the PL decay.*

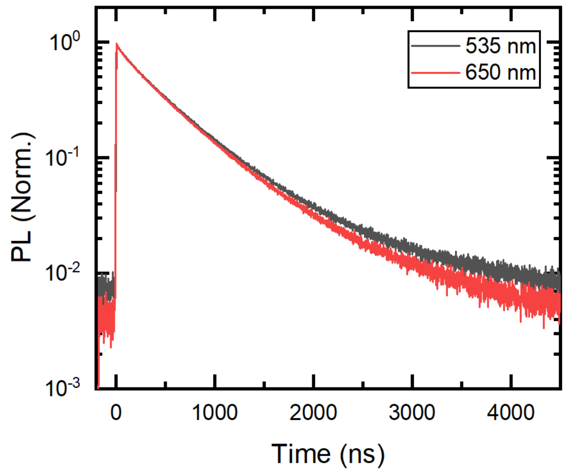

Supplementary Figure 16. *Normalised IR Transient PL Kinetics. The PbS-OA:TIPS-Tc film was excited at 650 nm (2.3 µW, 10 nJ/cm²) and 535 nm (4.0 µW, 15 nJ/cm²), at 0.2 MHz repetition rate. Contribution to the detected counts by background counts was removed before normalisation to the initial value of the PL decay.*

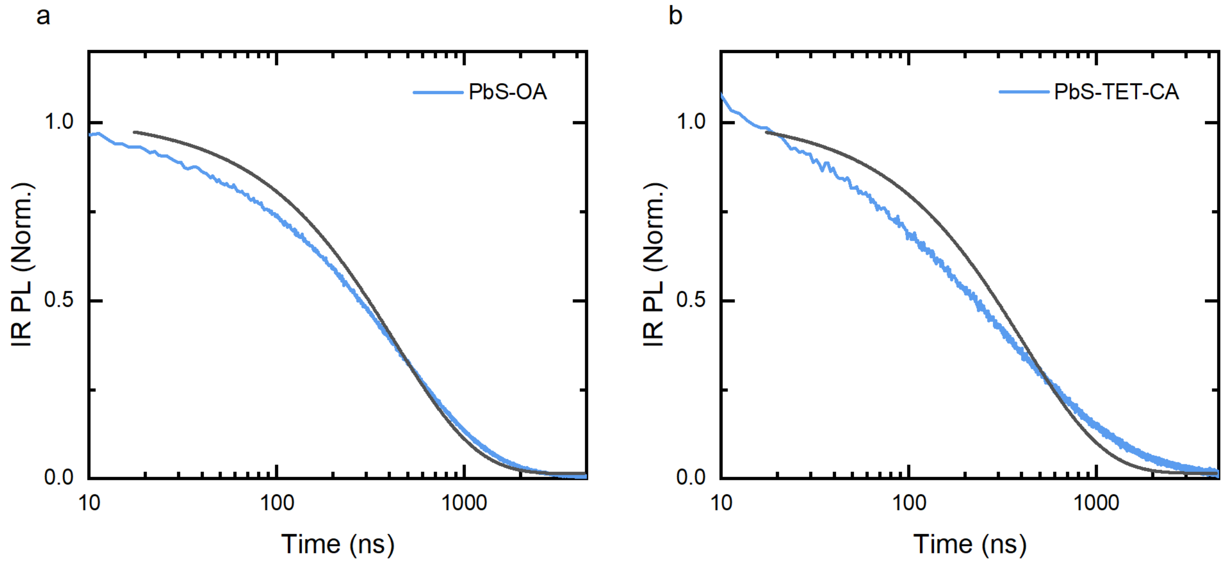

***Supplementary Figure 17**. Normalised IR transient PL kinetics for films of a) PbS-OA:TIPS-Tc and b) PbS-TET-CA:TIPS-Tc excited at 650 nm with fluence 10 nJ/cm^2, 0.2 MHz repetition rate (QD preferentially excited). The PL decay was fitted with a single exponential decay with decay rates 2.3 ± 0.2 μs^{-1} and 2.5 ± 0.2 μs^{-1} respectively.*

8.5 Triplet Flux Deconvolution

The flux (assigned to triplet transfer) into the PbS QD, $\phi_T(t)$, is found by deconvolving the intrinsic QD response $h(t)$ (650 nm excitation, the QDs impulse response) from the observed QD response with triplet transfer $y(t)$ (535 nm excitation). Here the ansatz is that the QD dynamics can be related to the intrinsic response as follows,[25]

$$y(t) = h(t) * \left(\delta(t) + \phi_T(t)\right). \quad \text{(S11)}$$

Where $\delta(t)$ is a delta function representing the fraction of photons in the 535 nm pump pulse that excites the QDs directly. To achieve appropriate levels of signal to noise, we perform a post-processing step where the $y(t)$ and $h(t)$ time series are binned, taking the average of 40 data points and reducing it to 1 data point respectively (at the mean time of the 40 data points). The deconvolution is calculated using a fast Fourier transform (FFT) as described by,

$$\delta(t) + \phi_T(t) = FFT^{-1}\left[\frac{FFT[y(t)](\omega)}{FFT[h(t)](\omega)}\right](t). \quad \text{(S12)}$$

The first 2 two data points after the pump excitation were removed (removing $\delta(t)$) giving the triplet flux into the PbS QDs (Supplementary Figure 18b). This triplet flux is then convolved with the intrinsic QD decay $h(t)$ to give the QD IR PL (directly proportional to the QD population) that is due to triplet transfer (Supplementary Figure 18c). The triplet flux shows unexpected behaviour, where it rises over the first ~500 ns after the pump pulse. In previous measurements on bilayers of tetracene and PbS QDs, the deconvolution of the QD PL showed a triplet flux that doesn't rise at all after the pump excitation and only decays over a μs time scale.[25]

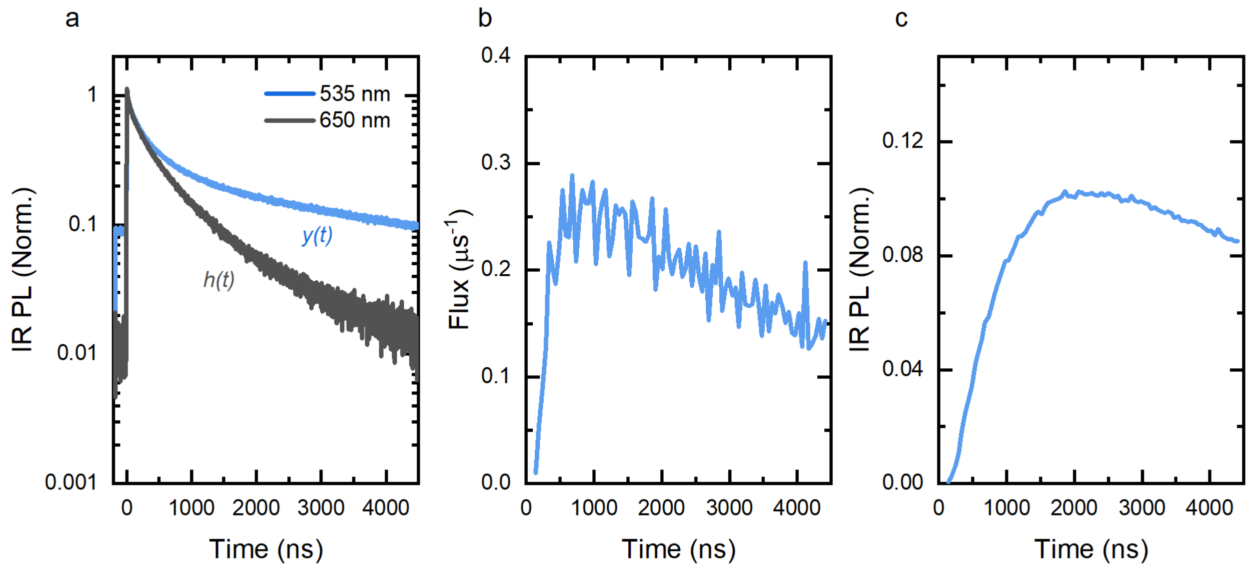

***Supplementary Figure 18**. a) PbS-TET-CA:TIPS-Tc transient IR PL for the PMF under intrinsic decay (650 nm excitation) and triplet transfer (535 nm excitation), at 0.2 MHz repetition rate. b) Deconvoluted excitation flux into the PbS QD). c) Triplet flux convolved with the intrinsic QD decay (h(t)) to give the PbS PL resulting from purely triplet transfer.*

8.6 Triplet Transfer Models

Here we discuss possible kinetics schemes for the triplet transfer between TIPS-Tc and PbS QDs and compare the dynamics to the IR transient PL measurements. We propose two kinetic models, the first requires only two species, the TIPS-Tc triplet $[T_{1,1}]$ and the excited QD state $[QD_1]$; the second introduces a third intermediate species $[I]$.

8.6.1 Two-Species Model

In this kinetic scheme we assume there are only two states participating in the triplet transfer, the TIPS-Tc triplet state $[T_{1,1}]$ and the excited PbS QD state $[QD_1]$. The dynamics of this system are described as follows,

$$\frac{d[T_{1,1}]}{dt} = -(k_1 + k_{TET})[T_{1,1}] - k_2[T_{1,1}]^2 + \eta_{SF} \cdot G_T(z)\,, \quad \text{(S13)}$$

$$\frac{d[QD_1]}{dt} = -k_{QD}[QD_1] + k_{TET}[T_{1,1}]\,, \quad \text{(S14)}$$

with rates as described earlier. To simplify this system of differential equations we assume the case of the low $[T]$ limit where contribution by the $k_2[T]^2$ can be ignored and that triplet transfer out-competes triplet intrinsic decay ($k_1 + k_{TET} \sim k_{TET}$). Solving this system leads to a triplet population given by,

$$[T_{1,1}]\,(t) = [T_{1,1}]_0 e^{-k_{TET}t}, \quad \text{(S15)}$$

where $[T_{1,1}]_0$ is the initial triplet density after singlet fission. The triplet flux into the QD is,

$$\phi_T(t) = k_{TET}[T_{1,1}](t) = k_{TET}[T_{1,1}]_0 e^{-k_{TET}t}. \quad \text{(S16)}$$

The QD population due to this transfer is given by,

$$[QD_1]_\phi(t) = -\frac{k_{TET}[T_{1,1}]_0}{k_{TET}-k_{QD}}\left(e^{-k_{TET}t} - e^{-k_{QD}t}\right). \quad (S17)$$

While the QD population due to direct excitation (650 nm excitation) is,

$$[QD_1]_{PL}(t) = [QD_1]_0 e^{-k_{QD}t}\,, \quad (S18)$$

where $[QD_1]_0$ is the initial excited QD population. This set of equations allows for simultaneous calculation of the QD intrinsic PL decay $[QD_1]_{PL}(t)$, the triplet flux into the QD $\phi_T(t)$ and QD population due to transfer $[QD_1]_\phi(t)$. Supplementary Figure 19 shows the best achieved global fitting of these functions to the measured values. The quality of this fit is very poor, showing large systematic discrepancies of the observed trends. Notably, the triplet flux does rise over the first ~500 ns as observed in the measured data and the QD population from transfer peaks and falls faster than measured. We constrain the system such that the triplet transfer rate is the same as given by the ns-TA measurements. The value for the QD intrinsic decay rate k_{QD} is slightly smaller than the values measured by the fitting of the QD GSB in ns-TA and the observed transient PL decay (Table S5).

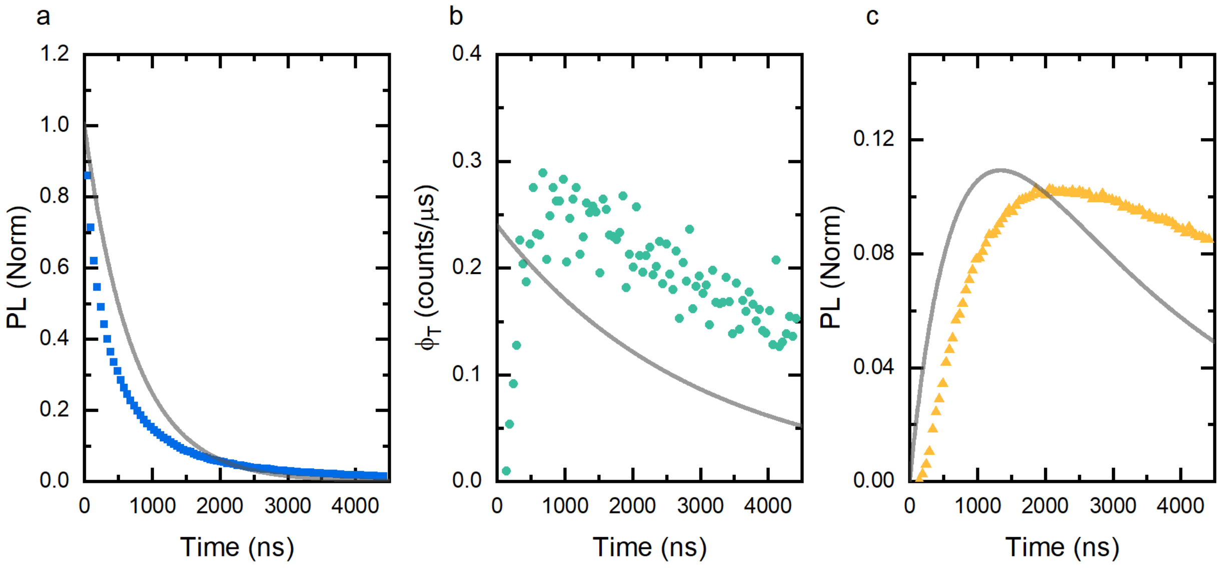

Supplementary Figure 19. *Two species model fitting of the IR transient PL for a PbS-TET-CA:TIPS-Tc film. The intrinsic QD PL decay, triplet flux into the QD and QD PL counts from triplet transfer, was fitted globally. a) PbS-TET-CA:TIPS-Tc intrinsic QD PL decay (650 nm excitation). b) Triplet flux into the PbS QD in a film of PbS-TET-CA:TIPS-Tc after 535 nm excitation of the SF-host. c) PbS-TET-CA:TIPS-Tc QD PL resulting from triplet transfer (after excitation with 535 nm), calculated by convolution of the triplet flux and the PbS-TET-CA intrinsic decay dynamics.*

Table S5. Comparison of the triplet transfer kinetic parameters for a two-species model with global fitting to the intrinsic QD decay, triplet flux and QD PL from triplet transfer.

k_{TET} (1/μs)	k_{QD} (1/μs)	k_{QD} (1/μs)	k_{QD} (1/μs)
TA fit	TA fit	TrPL fit	Transfer TrPL fit
0.34 ± 0.03	3.3 ± 0.2	2.5 ± 0.2	1.4 ± 0.1

Supplementary Figure 20 shows the best achieved two species fitting against the measured QD PL from transfer alone (not globally fitted). The fitted kinetic for the transient PL from triplet transfer is reasonable, showing a lower discrepancy with the measured response. However, the corresponding kinetics for the QD intrinsic decay and triplet flux show considerable deviation from the data. This fitting method requires a significantly slower QD decay relative to the values measured by nsTA and transient PL alone (Table S6). This discrepancy between QD decay rate extracted by the QD population from triplet transfer (535 nm excitation) and the rate obtained by optical excitation of the QDs directly (650 nm excitation) is consistent with the hypothesis that there exist two subsets of QDs within the film. One set that is affected by QD aggregation to a greater extent, resulting in short QD lifetimes due to trapping and lower triplet transfer due to the separation of triplet donor and acceptor. The other subset of QDs are isolated within the SF-host having slower decay (similar to the rate measured for an isolated dot in solution ~0.5 μs^{-1}) and high triplet transfer due to the maximal interaction between donor and acceptor. The rise in triplet flux could be an artefact in this case as the ansatz in equation S11 would not be valid. We leave the investigation of this hypothesis to future work.

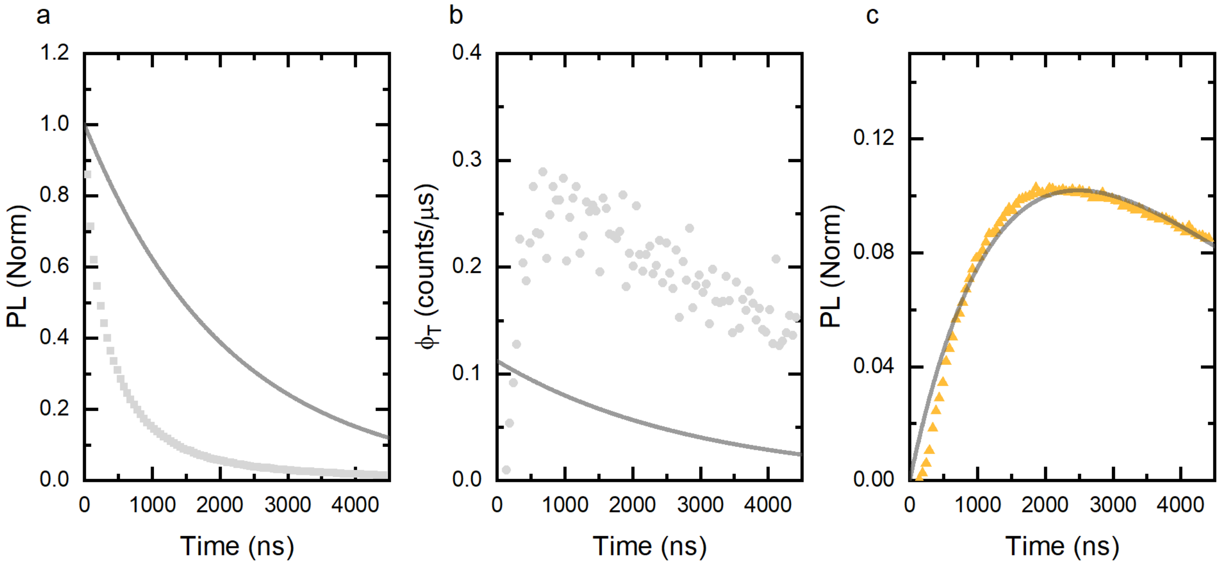

***Supplementary Figure 20**. Two species model fitting of the IR transient PL for a PbS-TET-CA:TIPS-Tc film. Only data for QD PL from transfer was fitted and the required intrinsic QD PL decay, triplet flux into the QD plotted beside the measured counts. a) PbS-TET-CA:TIPS-Tc intrinsic QD PL decay (650 nm excitation). b) Triplet flux into the PbS QD in a film of PbS-TET-CA:TIPS-Tc after 535 nm excitation of the SF-host. c) PbS-TET-CA:TIPS-Tc QD PL resulting from triplet transfer (after excitation with 535 nm), calculated by convolution of the triplet flux and the PbS-TET-CA intrinsic decay dynamics.*

Table S6. *Comparison of the triplet transfer kinetic parameters for a two-species model with the fitting of the QD PL from transfer alone.*

k_{TET} (1/μs)	k_{QD} (1/μs)	k_{QD} (1/μs)	k_{QD} (1/μs)
TA fit	TA fit	TrPL fit	Transfer TrPL fit
0.34 ± 0.03	3.27 ± 0.15	2.5 ± 0.2	0.50 ± 0.05

8.6.2 Three-Species Model

In this kinetic scheme, we assume the existence of an intermediate state $[I]$ participating in the triplet transfer. The dynamics of this system are described as follows,

$$\frac{d[T_{1,1}]}{dt} = -(k_1 + k_{TET_1})[T_{1,1}] - k_2[T_{1,1}]^2 + \eta_{SF} \cdot G_T(z)\,, \qquad (S19)$$

$$\frac{d[I]}{dt} = -k_{TET_2}[I] + k_{TET_1}[T_{1,1}]\,, \qquad (S20)$$

$$\frac{d[QD_1]}{dt} = -k_{QD}[QD_1] + k_{TET_2}[I]\,, \qquad (S21)$$

where k_{TET_1} is the triplet transfer rate from the TIPS-Tc to the intermediate (this rate is the same as the previously discussed k_{TET} as it quantifies triplet loss from the TIPS-Tc) and k_{TET_2} is the rate of triplet transfer from the intermediate to the QD excited state. To simplify this system of differential equations we assume the case of the low $[T]$ limit where contribution by the $k_2[T]^2$ can be ignored and that triplet transfer out-competes triplet intrinsic decay ($k_1 + k_{TET_1} \sim k_{TET_1}$) and there is 100% transfer from the intermediate to the QD). Solving this system leads to a triplet population given by,

$$[T_{1,1}](t) = [T_{1,1}]_0 e^{-k_{TET_1} t}, \qquad (S22)$$

where $[T_{1,1}]_0$ is the initial triplet density after singlet fission. The intermediate state population is given by,

$$[I](t) = -\frac{k_{TET_1}[T_{1,1}]_0}{k_{TET_1} - k_{TET_2}}\left(e^{-k_{TET_1} t} - e^{-k_{TET_2} t}\right), \qquad (S23)$$

where we assume the initial population of the intermediate state is $[I](0) = 0$. The triplet flux into the QD is,

$$\phi_T(t) = k_{TET_2} I(t) = -\frac{k_{TET_1} k_{TET_2}[T_{1,1}]_0}{k_{TET_1} - k_{TET_2}}\left(e^{-k_{TET_1} t} - e^{-k_{TET_2} t}\right), \qquad (S24)$$

The QD population due to this transfer is given by,

$$[QD_1]_\phi(t) = \frac{k_{TET_1} k_{TET_2}[T_{1,1}]_0\left(k_{QD}\left(e^{-k_{TET_2} t} - e^{-k_{TET_1} t}\right) + k_{TET_2}\left(e^{-k_{TET_1} t} - e^{-k_{QD} t}\right) + k_{TET_1}\left(e^{-k_{QD} t} - e^{-k_{TET_2} t}\right)\right)}{(k_{TET_1} - k_{TET_2})(k_{TET_1} - k_{QD})(k_{TET_2} - k_{QD})}, \qquad (S25)$$

While the QD population due to direct excitation (650 nm excitation) is,

$$[QD_1]_{PL}(t) = [QD_1]_0 e^{-k_{QD} t}\,, \qquad (S26)$$

where $[QD_1]_0$ is the initial excited QD population. This set of equations allows for simultaneous calculation of the QD intrinsic PL decay $[QD_1]_{PL}(t)$, the triplet flux into the QD $\phi_T(t)$ and QD population due to transfer $[QD_1]_{\phi}(t)$. Supplementary Figure 21 shows the best achieved global fitting of these functions to the measured values. We constrain the system such that the triplet transfer rate k_{TET_1} is the same as given by the ns-TA measurements. The agreement between measured values and fit is the strongest out of the three investigated fitting procedures, reproducing the observed rises and falls in the various time-dependent quantities. To accurately fit the rise in the triplet flux requires the k_{TET2} fitting parameter. This introduction of an intermediate state is not arbitrary as it has been shown that the TET-CA ligand is crucial to the triplet transfer process in solution and its rate of transfer into the PbS QD has been calculated.[27] Thus we assign this intermediate state as the TET-CA triplet $[I] = [T_{1,2}]$.[27] Supplementary Figure 11d summarises the transfer dynamics where the TIPS-Tc triplet is transferred to an intermediate state with rate k_{TET1}, followed by a second triplet transfer with rate k_{TET2} to the PbS quantum dot. For illustrative purposes, the TET-CA triplet and TIPS-Tc triplet populations in Supplementary Figure 11d, have been smoothed with using the Savitzky-Golay method with a 2nd order polynomial fitting over a 10 and 7 data point window respectively.

Table S7 compares the various kinetic parameters. The value for the QD intrinsic decay rate k_{QD} is again slightly smaller than the values measured by the fitting of the QD GSB in ns-TA and the observed transient PL decay. This might be to the two subset hypothesis mentioned previously.

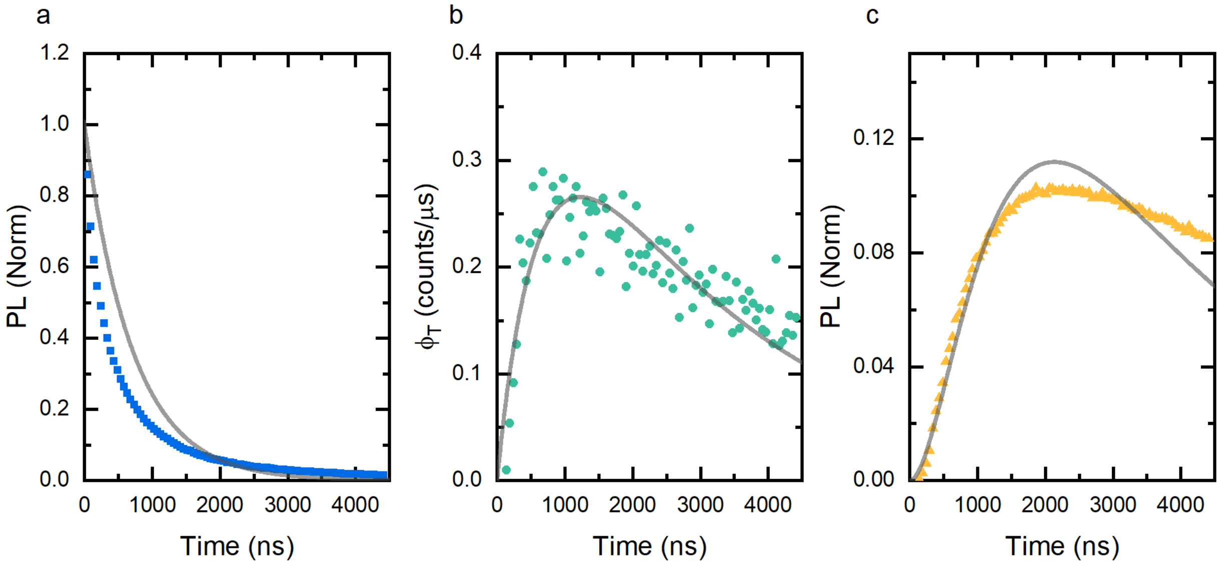

Supplementary Figure 21. *Three species model fitting of the IR transient PL for a PbS-TET-CA:TIPS-Tc film. The intrinsic QD PL decay, triplet flux into the QD and QD PL counts from triplet transfer, was fitted globally. a) PbS-TET-CA:TIPS-Tc intrinsic QD PL decay (650 nm excitation). b) Triplet flux into the PbS QD in a film of PbS-TET-CA:TIPS-Tc after 535 nm excitation of the SF-host. c) PbS-TET-CA:TIPS-Tc QD PL resulting from triplet transfer (after excitation with 535 nm), calculated by convolution of the triplet flux and the PbS-TET-CA intrinsic decay dynamics.*

Table S7. *Comparison of the triplet transfer kinetic parameters for a three-species model with global fitting to the intrinsic QD decay, triplet flux and QD PL from triplet transfer. The monomolecular TIPS-Tc triplet decay rate k_1 and TIPS-Tc to PbS-TET-CA triplet transfer rate k_{TET1} are measured by fitting to the ns-TA.*

k_{TET1} (1/μs)	k_{TET2} (1/μs)	k_{QD} (1/μs)	k_{QD} (1/μs)	k_{QD} (1/μs)	k_1 (1/μs)
TA fit	ϕ_T(t) fit	TA fit	TrPL fit	Transfer TrPL fit	TA fit
0.34 ± 0.03	1.6 ± 0.1	3.27 ± 0.15	2.5 ± 0.2	1.40 ± 0.2	(1.05 ± 0.10)x10^{-2}

9 Upconversion

9.1 Energy Levels of Anthrathiophenes

To get an idea of the triplet energies of the thienoanthracenes we attached the TA-CA ligand to PbS QDs of different band gap and monitored the QD PL quenching. As can be seen in Supplementary Figure 22 significant quenching of ~50% is observed for QDs of bandgap 1.3 eV. Even greater quenching is observed for 1.4 eV QDs (~75%).

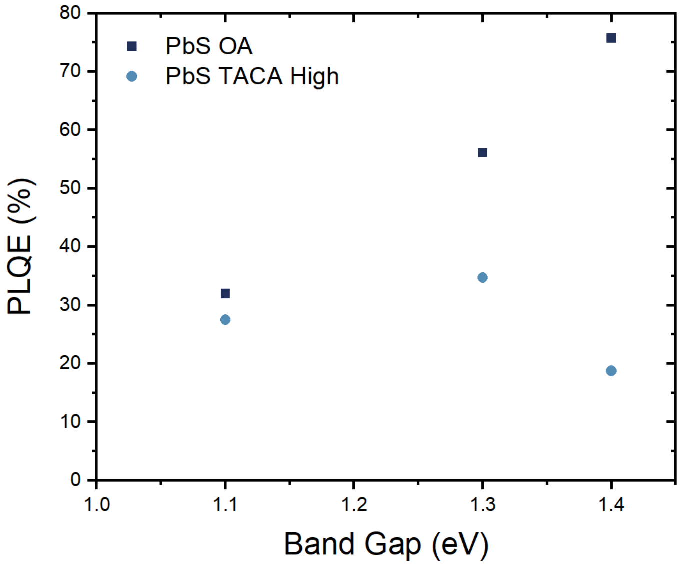

Supplementary Figure 22*. Photoluminescence quantum efficiency (PLQE) of PbS QDs of different bandgap with oleic acid (OA) or TA-CA ligands, excited at 658 nm.*

Compared to TA-CA the F-TA annihilator shows slightly higher lying singlet energies (Supplementary Figure 23). Likely the carboxylic acid group conjugates with the thienoanthracene core, extending the conjugation and lowering both the singlet and triplet energies of TA-CA relative to F-TA.

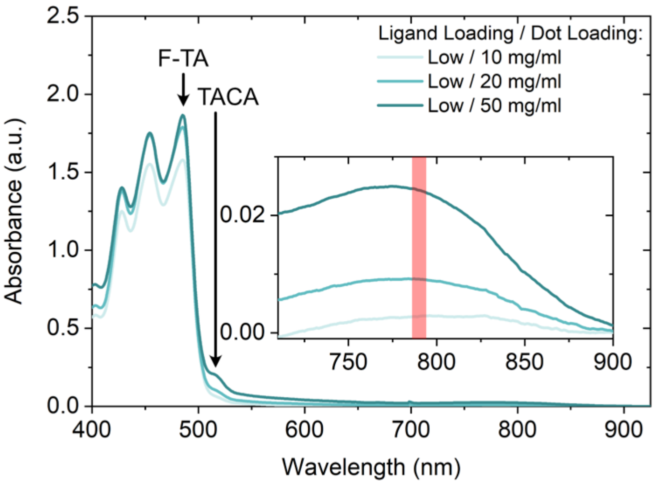

Supplementary Figure 23. Absorption of F-TA films with different loadings of PbS TA-CA QDs. Arrows show the first vibronic peak of the absorption of F-TA and TA-CA.

9.2 Triplet Energy transfer in solution

To better understand the triplet transfer dynamics in the TTA-UC system we use ps- and ns-TA. As can be seen in Supplementary Figures 24 and 25, when the TA-CA ligand is attached the QD ground state bleach recovers faster following a bi-exponential decay with time constants of 23.2 ps and 57.5 ns. As the QD ground state bleach decays a new feature at 530 – 630 nm grows in, Supplementary Figure 25. As no signal from the ligand triplet is observed on the ps timescale the faster decay component (23.2 ps) is likely related to surface trapping due to traps introduced during the ligand exchange. The slower time component matches the time component observed for the rise in the ligand triplet signal (64.1 ns), hence corresponding to the rate of triplet transfer from QD to ligand.

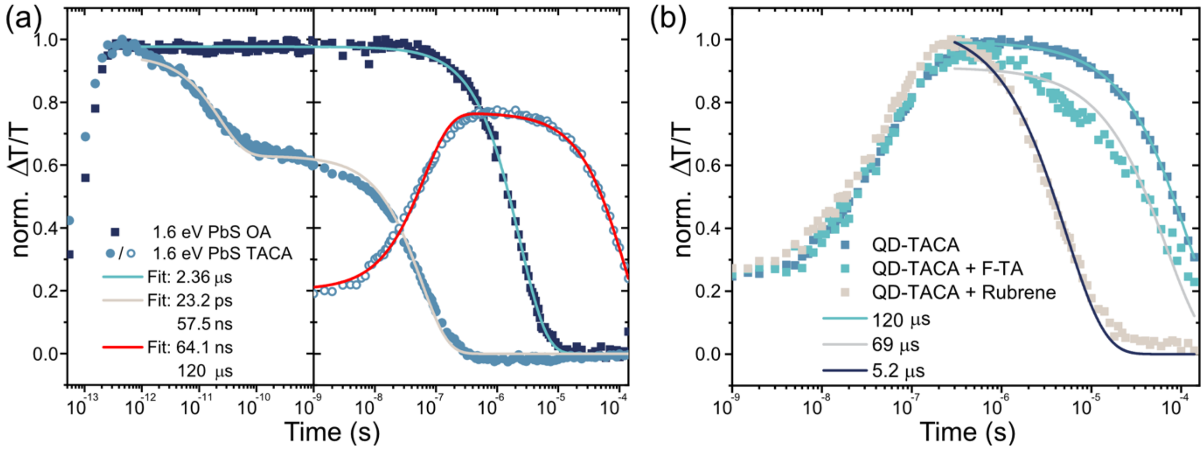

Supplementary Figure 24. *(a) Combined ps and ns transient absorption kinetics of as synthesized oleic acid capped PbS QDs (PbS OA) and TA-CA ligand exchanged PbS QDs (PbS TA-CA). The QD exciton bleach kinetics (filled markers) is followed at 780 nm, and the TA-CA ligand triplet kinetics (open circles) is followed at 600 nm. (b) ns-transient absorption kinetics of the PbS bound TA-CA ligand triplet in the absence and presence of F-TA or Rubrene annihilators.*

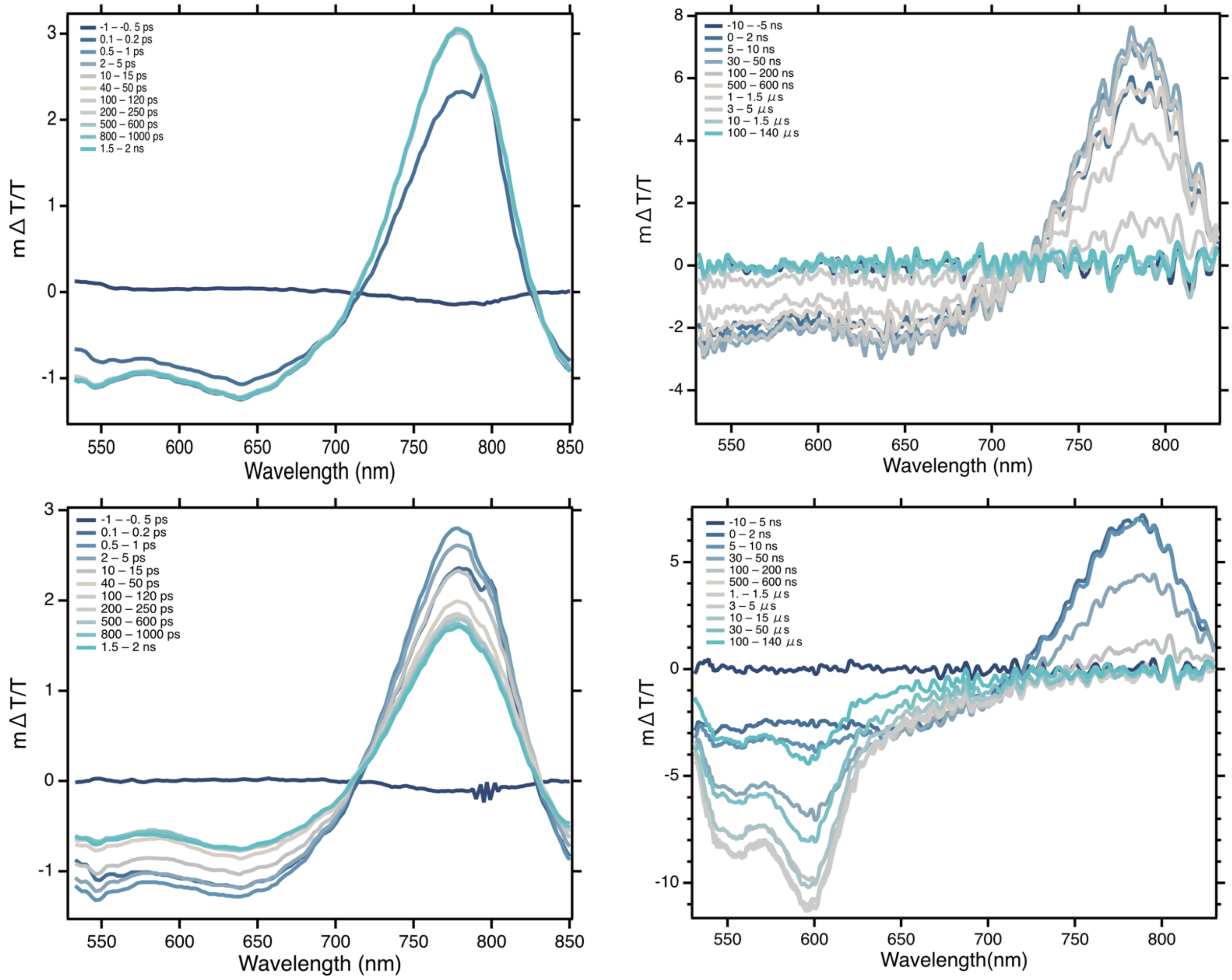

Supplementary Figure 25. *ps-TA absorption spectra (left) and ns-TA absorption spectra (right) of PbS OA QDs (top) and PbS TA-CA QDs with low ligand loading (bottom). The ligand triplet absorption appears around 550-600 nm in the ns-TA data.*

The ligand triplet decays with a time constant of 120 µs. When F-TA or rubrene is added to the solution the ligand triplet is quenched, Supplementary Figure 24b, indicating triplet energy transfer to the annihilator. The quenching by F-TA is rather inefficient (~40%), a consequence of the similar triplet energies of the TA-CA and F-TA molecules (see below). In further support of this, the quenched TA-CA triplet decay cannot be properly reproduced by a single exponential decay suggesting forward and back transfer between F-TA and TA-CA is possible (Supplementary Figure 24b). Rubrene, with a significantly lower triplet energy of 1.14 eV,[28] rapidly quenches the TA-CA triplet, achieving close to quantitative (96%) triplet energy transfer. However, the difference in TA-CA triplet quenching by rubrene and F-TA cannot explain the significantly lower UC intensity for the F-TA UC samples discussed below. It would suggest that F-TA also has shortcomings as an annihilator, perhaps the bulky TIBS groups prevent two molecules from achieving an optimal geometry for efficient TTA in solution.

9.3 Upconversion in solution

To determine if PbS-TA-CA QDs are suitable for photon upconversion applications the triplet sensitization and upconversion properties are first studied in solution. The absorption spectra of PbS QDs ligand exchanged with TA-CA ligands are shown in Supplementary Figure 26. Three different degrees of ligand coverage were investigated. With the highest degree of coverage, a relatively large change in the QD excitonic absorption peak can be observed relative to the OA-ligated QDs. Such changes can either arise from a large electronic interaction between the ligand and QD, or if the QD surface is depleted during the ligand exchange leading to a smaller PbS core. For the medium and low ligand coverage the excitonic peak is preserved. Furthermore, upconversion is less efficient for the sensitizers with the highest ligand coverage (Supplementary Figure 26b) suggesting that more surface traps are present for the higher degree of ligand exchange. We therefore chose to use the low coverage PbS-TA-CA QDs as sensitizer for our further studies, from now referred to PbS-TA-CA. The upconversion quantum yield for concentrated rubrene solutions (10 mM) with 25 mg/ml PbS-TA-CA sensitizers is 0.7%, determined in an integrating sphere. The upconversion quantum yield is on par, but on the low side of what has been reported for other unshelled PbS QD-tetracene sensitizers.[29,30] Upconverted emission was faintly observed when F-TA was used as an annihilator in solution, however, the quantum yield was too low to determine on our setup.

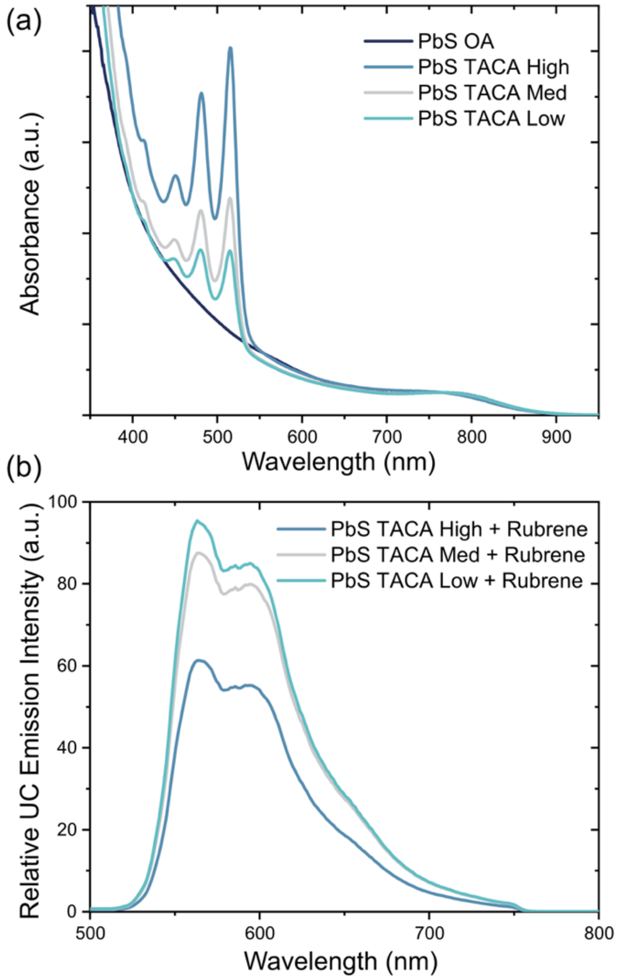

***Supplementary Figure 26**. (a) Absorption spectrum of PbS QDs before and after ligand exchange with TACA ligands. (b) Relative photon upconversion emission of solution samples containing 25mg/ml PbS-*

TACA sensitizers with different ligand coverage and 10 mM Rubrene in toluene. Excitation at 790 nm (10 W/cm²) and emission recorded at front face geometry using a 750 short pass filter to remove laser scatter.

9.4 Upconversion in Film

At low excitation intensities the upconverted emission depends quadratically on excitation intensity, whereas at sufficiently high excitation intensities this dependence becomes linear. In the quadratic regime, the main triplet decay pathway is intrinsic whereas in the linear regime, triplet decay is dominated by bimolecular TTA. A crossover point where intrinsic decay and decay *via* TTA are equal is referred to as the threshold intensity, I_{th}. The threshold intensity can be estimated according to Equation S27: [31–34]

$$I_{th} = \frac{k_T^2}{k_{TTA}\alpha[S]} \qquad \text{(S27)}$$

where k_T is the intrinsic triplet decay rate of the annihilator, k_{TTA} is the bimolecular triplet-triplet annihilation rate, α is the absorption crossection of the sensitizer and $[S]$ is the concentration of the sensitizer. I_{th} was determined graphically as shown in Figure 4b.

The fluorescence quantum yield (QY) of neat F-TA was measured in an integrating sphere to 17.8±1.1%, Table S8. However, with the low absorption of the PbS QDs in the 10 mg/ml loaded films, integrating sphere measurements to determine the upconversion quantum yield (UCQY) are inappropriate. Instead, we compared the emission when exciting the film at 790 nm and 405 nm and estimated UCQY from the relative emission intensity, absorption, and excitation intensity. An average UCQY of 0.1±0.06 % was estimated in this way, Table S8.

***Table S8**. Optical properties of F-TA films with PbS-TA-CA sensitizer QDs with low ligand loading.*

	10 mg/ml
A_{790nm}	0.0038±0.0014[a]
A_{405nm}	0.282±0.018[a]
QY	17.8±1.1 %[b,c]
UCQY	0.1±0.06 %[b,d]
I_{th} (W/cm²)	1.60

a) Average from 2 films. b) Average from 2 films measured at 4 spots each. c) measured with an integrating sphere. d) relative measurement compared to 405 nm excitation of the same film.

10 References

1. Heenan, R. K. *et al.* Small angle neutron scattering using sans2d. *Neutron News* **22**, 19–21 (2011).
2. https://www.isis.stfc.ac.uk/. *STFC ISIS Neutron and Muon Source Home Page* (2019).
3. http://www.mantidproject.org/. *Mantid Project Home Page* (2019).
4. Wignall, G. D. & Bates, F. S. Absolute calibration of small-angle neutron scattering data. *Journal of Applied Crystallography* **20**, 28–40 (1987).

5. Weir, M. P. *et al.* Ligand Shell Structure in Lead Sulfide–Oleic Acid Colloidal Quantum Dots Revealed by Small-Angle Scattering. *The Journal of Physical Chemistry Letters* **10**, 4713–4719 (2019).
6. http://www.sasview.org/. *SasView Hompeage* (2019).
7. Percus, J. K. & Yevick, G. J. Analysis of Classical Statistical Mechanics by Means of Collective Coordinates. *Phys. Rev.* **110**, 1–13 (1958).
8. Jiang, Z. GIXSGUI: a MATLAB toolbox for grazing-incidence X-ray scattering data visualization and reduction, and indexing of buried three-dimensional periodic nanostructured films. *J Appl Cryst* **48**, 917–926 (2015).
9. Pospelov, G. *et al.* BornAgain: software for simulating and fitting grazing-incidence small-angle scattering. *J Appl Cryst* **53**, 262–276 (2020).
10. Hines, M. A. & Scholes, G. D. Colloidal PbS Nanocrystals with Size-Tunable Near-Infrared Emission: Observation of Post-Synthesis Self-Narrowing of the Particle Size Distribution. *Advanced Materials* **15**, 1844–1849 (2003).
11. Zhang, J. *et al.* Synthetic Conditions for High-Accuracy Size Control of PbS Quantum Dots. *Journal of Physical Chemistry Letters* **6**, 1830–1833 (2015).
12. Moreels, I. *et al.* Size-Dependent Optical Properties of Colloidal PbS Quantum Dots. (2009) doi:10.1021/nn900863a.
13. Mello, J. de, Wittmann, H. & Friend, R. An improved experimental determination of external photoluminescence quantum efficiency. *Advanced materials* **9**, 230–232 (1997).
14. Allardice, J. R. *et al.* Engineering Molecular Ligand Shells on Quantum Dots for Quantitative Harvesting of Triplet Excitons Generated by Singlet Fission. *Journal of the American Chemical Society* **141**, 12907–12915 (2019).
15. Tabachnyk, M. *et al.* Resonant energy transfer of triplet excitons from pentacene to PbSe nanocrystals-Supplementary Information. *Nature Materials* **13**, 1033–1038 (2014).
16. Allardice, J. R. *et al.* Engineering Molecular Ligand Shells on Quantum Dots for Quantitative Harvesting of Triplet Excitons Generated by Singlet Fission. *Journal of the American Chemical Society* **141**, 12907–12915 (2019).

17. Tang, M. L., Reichardt, A. D., Okamoto, T., Miyaki, N. & Bao, Z. Functionalized Asymmetric Linear Acenes for High-Performance Organic Semiconductors: Acenes in High-Performance Organic Semiconductors. *Adv. Funct. Mater.* **18**, 1579–1585 (2008).

18. Schnürch, M., Spina, M., Khan, A. F., Mihovilovic, M. D. & Stanetty, P. Halogen dance reactions—A review. *Chem. Soc. Rev.* **36**, 1046–1057 (2007).

19. Huang, H. *et al.* BN-embedded eleven-ring fused heteroaromatics: Synthesis, optoelectronic properties and fluoride susceptibility. *Dyes and Pigments* **177**, 108271 (2020).

20. Tang, M. L., Reichardt, A. D., Siegrist, T. & Mannsfeld, S. C. B. Trialkylsilylethynyl-Functionalized Tetraceno[2,3-b]thiophene and Anthra[2,3-b]thiophene Organic Transistors. 8.

21. Toolan, D. T. W. *et al.* Mixed Small-Molecule Matrices Improve Nanoparticle Dispersibility in Organic Semiconductor-Nanoparticle Films. *Langmuir* **39**, 4799–4808 (2023).

22. Shih, J.-L., Jansone-Popova, S., Huynh, C. & May, J. A. Synthesis of azasilacyclopentenes and silanols via Huisgen cycloaddition-initiated C–H bond insertion cascades. *Chem. Sci.* **8**, 7132–7137 (2017).

23. De Mello, J. C., Wittmann, H. F. & Friend, R. H. An improved experimental determination of external photoluminescence quantum efficiency. *Advanced Materials* **9**, 230–232 (1997).

24. Thompson, N. J. *et al.* Energy harvesting of non-emissive triplet excitons in tetracene by emissive PbS nanocrystals. *Nature Materials* **13**, 1039–1043 (2014).

25. Thompson, N. J. *et al.* Energy harvesting of non-emissive triplet excitons in tetracene by emissive PbS nanocrystals. *Nature Materials* **13**, 1039–1043 (2014).

26. Stern, H. L. *et al.* Vibronically coherent ultrafast triplet-pair formation and subsequent thermally activated dissociation control efficient endothermic singlet fission. *Nature Chemistry* **9**, 1205–1212 (2017).

27. Gray, V. *et al.* Direct vs Delayed Triplet Energy Transfer from Organic Semiconductors to Quantum Dots and Implications for Luminescent Harvesting of Triplet Excitons. *ACS Nano* 10.1021/acsnano.9b09339 (2020) doi:10.1021/acsnano.9b09339.

28. Herkstroeter, W. G. & Merkel, P. B. THE TRIPLET STATE ENERGIES OF RUBRENE AND DIPHENYLISO-BENZOFURAN. *Journal of Photochemistry* **16**, 331–341 (1981).

29. Huang, Z., Simpson, D. E., Mahboub, M., Li, X. & Tang, M. L. Ligand enhanced upconversion of near-infrared photons with nanocrystal light absorbers. *Chem. Sci.* **7**, 4101–4104 (2016).

30. Huang, Z. *et al.* PbS/CdS Core–Shell Quantum Dots Suppress Charge Transfer and Enhance Triplet Transfer. *Angewandte Chemie - International Edition* **56**, 16583–16587 (2017).

31. Monguzzi, A., Mézyk, J., Scotognella, F., Tubino, R. & Meinardi, F. Upconversion-induced fluorescence in multicomponent systems: Steady-state excitation power threshold. *Physical Review B - Condensed Matter and Materials Physics* **78**, 195112 (2008).

32. Haefele, A., Blumhoff, J., Khnayzer, R. S. & Castellano, F. N. Getting to the (Square) root of the problem: How to make noncoherent pumped upconversion linear. *Journal of Physical Chemistry Letters* **3**, 299–303 (2012).

33. Auckett, J. E. *et al.* Efficient up-conversion by triplet-triplet annihilation. *Journal of Physics: Conference Series* **185**, (2009).

34. Dzebo, D., Börjesson, K., Gray, V., Moth-Poulsen, K. & Albinsson, B. Intramolecular Triplet-Triplet Annihilation Upconversion in 9,10-Diphenylanthracene Oligomers and Dendrimers. *Journal of Physical Chemistry C* **120**, 23397–23406 (2016).
